# Exploiting codon usage identifies intensity-specific modifiers of Ras/MAPK signaling *in vivo*

**DOI:** 10.1371/journal.pgen.1009228

**Published:** 2020-12-09

**Authors:** Jessica K. Sawyer, Zahra Kabiri, Ruth A. Montague, Scott R. Allen, Rebeccah Stewart, Sarah V. Paramore, Erez Cohen, Hamed Zaribafzadeh, Christopher M. Counter, Donald T. Fox

**Affiliations:** 1 Department of Pharmacology & Cancer Biology, Duke University School of Medicine, Durham, North Carolina, United States of America; 2 Department of Cell Biology, Duke University School of Medicine, Durham, North Carolina, United States of America; 3 Duke Cancer Institute, Duke University School of Medicine, Durham, North Carolina, United States of America; The University of North Carolina at Chapel Hill, UNITED STATES

## Abstract

Signal transduction pathways are intricately fine-tuned to accomplish diverse biological processes. An example is the conserved Ras/mitogen-activated-protein-kinase (MAPK) pathway, which exhibits context-dependent signaling output dynamics and regulation. Here, by altering codon usage as a novel platform to control signaling output, we screened the *Drosophila* genome for modifiers specific to either weak or strong Ras-driven eye phenotypes. Our screen enriched for regions of the genome not previously connected with Ras phenotypic modification. We mapped the underlying gene from one modifier to the ribosomal gene RpS21. In multiple contexts, we show that RpS21 preferentially influences weak Ras/MAPK signaling outputs. These data show that codon usage manipulation can identify new, output-specific signaling regulators, and identify RpS21 as an *in vivo* Ras/MAPK phenotypic regulator.

## Author summary

Cellular communication is critical in controlling the growth of organs and must be carefully regulated to prevent disease. The Ras signaling pathway is frequently used for cellular communication of tissue growth regulation but can operate at different signaling strengths. Here, we used a novel strategy to identify genes that specifically tune weak or strong Ras signaling states. We find that the gene RpS21 preferentially tunes weak Ras signaling states.

## Introduction

Conserved signal transduction pathways are employed throughout nature during diverse processes such as cell fate decisions and tissue growth. These same pathways can be aberrantly regulated in disease. Large numbers of molecular regulators of these pathways have been identified using high-throughput genetic screening. Additionally, quantitative imaging approaches have revealed intricate signaling regulation. This regulation includes feedback control of the duration or strength of a downstream biochemical signaling output (e.g., weak or strong activation of a target gene). A current challenge is to place the numerous identified signaling pathway regulators in the context of complex signaling dynamics, and to relate such regulation to *in vivo* signal-dependent processes.

An example of the complexity of signaling regulation is the evolutionarily conserved Ras/mitogen activated protein kinase (MAPK) pathway [1]. In canonical MAPK signaling, receptor tyrosine kinase stimulation converts the Ras GTPase to an active GTP-bound conformation. Ras-GTP then activates the MAPK pathway, comprised of Raf kinases, which are activated by Ras and phosphorylate/active Mek kinases, which do the same to Erk kinases. Through highly successful modifier screen approaches in models such as the *Drosophila* eye [2–9] and *C*. *elegans* vulva [10–14], regulators of this core pathway have been identified. Additional *Drosophila* cell-based screens using a biochemical MAPK output (Erk phosphorylation) have identified many other Ras/MAPK regulators [15–17].

These numerous molecular regulators contribute to a diversity in Ras/MAPK signaling dynamics. Using an optogenetics-driven MAPK activation approach in cultured mammalian cells, it was revealed that distinct Ras/MAPK regulation (such as a paracrine STAT3 circuit) can distinguish between biochemical signaling outputs, namely sustained (strong) or transient (weak) Erk activation by Ras [18]. These biochemical outputs are regulated by negative feedback on Erk [19]. Importantly, *in vivo* context plays a role in whether a given strength of signaling output leads to a phenotypic output. Specifically, taking a similar optogenetic approach in the early developing fly embryo, it was shown that manipulating Erk activation strength has minimal effects on cells at the poles of the embryo, but has a profound impact on development of cells in the middle of the embryo [20]. Further, expressing an activating mutant of Mek in either *Drosophila* or zebrafish was recently shown to either activate or repress Erk phosphorylation depending on the cell type and gene expression environment [21]. The degree of Ras/MAPK signaling also plays a critical role in disease states. For example, altering the amount of Ras protein influences whether tumors develop in a carcinogenesis mouse model of lung cancer, or whether cancer cells mount a successful resistance response to chemotherapeutics [22,23]. These observations suggest that distinct Ras/MAPK regulation operates in distinct cellular contexts, and that this has biological consequences. Taken together, these studies highlight the need to better understand how distinct Ras/MAPK signaling states (e.g., strong or weak) are controlled by distinct sets of Ras/MAPK molecular regulators, in the context of an *in vivo* phenotype.

Here, we introduce a novel approach to genetically screen for signal output-specific regulators of Ras/MAPK signaling. This approach, which should be applicable to any signaling pathway, involves controlling the amount of active Ras protein produced by changing codon usage in the single *Drosophila Ras* gene [24] (FlyBase: *Ras85D*, hereafter *Ras)*. Rare codons are well-associated with poor mRNA translation [25]. Manipulating codon usage has been successfully employed in bacteria, for example as a means to tightly control the fatty acid synthesis pathway [26], and we previously demonstrated that changing rare codons in the mammalian Ras isoform *KRAS* to their common counterparts leads to elevated translation, protein, signaling, and transformation [27]. In this study, we report the generation and characterization of transgenic flies and cell lines whereby the amount of active Ras protein produced, the resultant level of Erk activation, and resultant rough-eye phenotype is dictated solely by the codon usage engineered into a given *Ras* transgene. We then report the use of such transgenic flies to screen a whole genome deficiency (termed *Df* for convenience) kit for genetic modifiers of eye phenotypes that are specific to only strong or only weak Ras/MAPK signaling. Our screen specifically looked for modifiers unique to specific Ras signaling states, by leveraging the differential signaling phenotypic output driven by rare versus common codons in the *Ras* gene. Importantly, the *Ras* gene enriched in rare codons used in our screen models more closely the rare codon-enriched sequence of human *KRAS* [27], which is the most frequently mutated RAS family member in human cancers [28].

Our screen enriched for genomic regions not previously ascribed to Ras phenotypic modification. Of the 15 *Dfs* identified, we successfully mapped the modification of *Df(2L)BSC692*, an enhancer of the rough-eye phenotype driven only by weak Ras/MAPK signaling, to the ribosomal protein S21 gene (*RpS21*). We show that RpS21 negatively regulates Ras protein levels in several contexts, the effect of which is preferentially manifested at low levels of MAPK signaling. This approach highlights the usefulness of codon manipulation as a viable approach to identify signal output-specific signaling regulation and introduces new genetic reagents to explore weak Ras signaling regulation in *Drosophila*. Our uniquely identified modifiers include those specific to Ras with rare codons, like that of human KRAS.

## Results

### Exploiting codon usage to control MAPK signaling output

**Fig 1:**
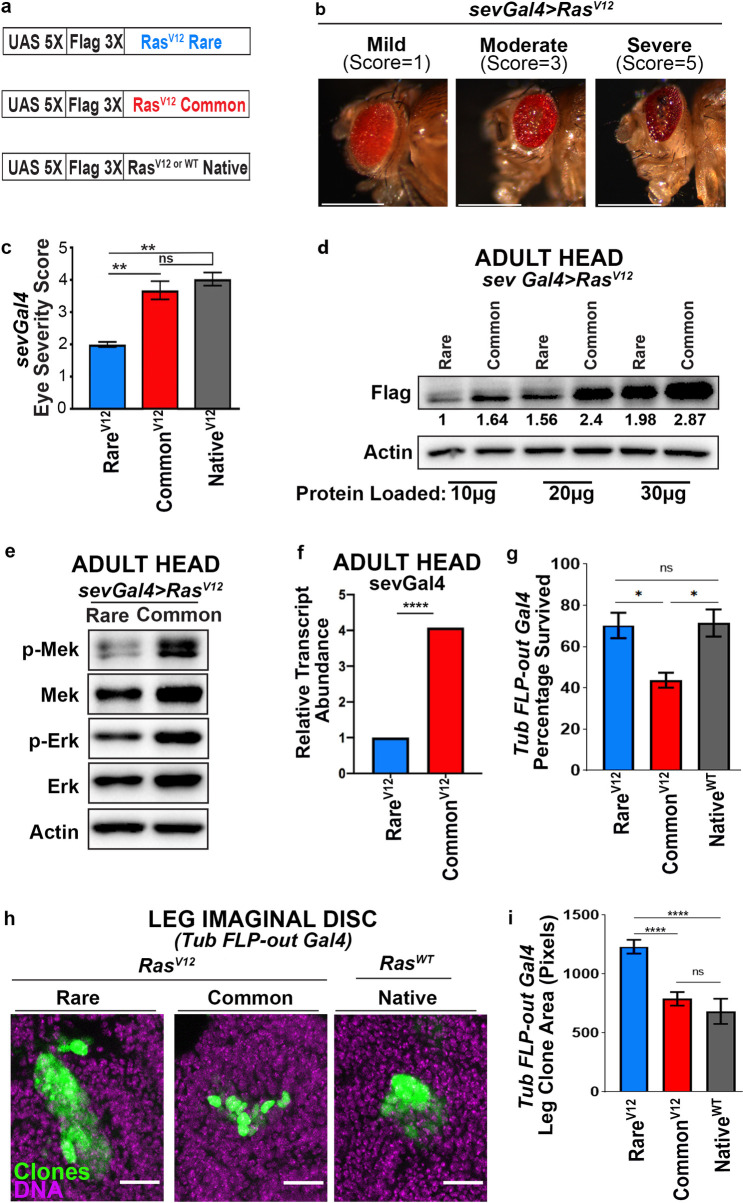
Exploiting codon usage to control MAPK signaling output. **(a)** Schematic representation of the FLAG epitope-tagged *Ras*^*V12*^ transgenes encoded by rare, common, or native codons. **(b)** Images representing the eye phenotypes assessed and the scoring system. Scale bars = 0.5mm. **(c)** The mean ± SEM eye severity score of the indicated *Ras*^*V12*^ transgenes from three replicate experiments at 25°C. Tukey’s multiple comparisons test was used for statistical comparisons. **(d)** Serial dilution western blot comparing protein levels of FLAG-Ras^V12^ common versus rare. 10, 20, and 30 ug of lysates derived from the heads of flies expressing the indicated versions of transgenic *Ras*^*V12*^ were immunoblotted with an anti-FLAG antibody. Bottom: quantification and protein loaded. **(e)** Immunoblot detection of phosphorylated (p-) and total Mek and Erk, and actin as a loading control from lysates derived from the head of flies with the indicated versions of transgenic *Ras*^*V12*^. The ratio of pErk/Erk and pMek/Mek for *Ras*^*V12*^ *Rare* is 0.89 and 0.94, respectively. The ratio of pErk/Erk and pMek/Mek for *Ras*^*V12*^ *Rare* is 1.43 and 2.05, respectively. (**f**) Quantitative RT-PCR, measured using 2^ΔΔCt, of animals expressing the indicated versions of transgenic *Ras*^*V12*^. Paired T-test. Data represent three independent replicates per condition, with 10–40 animals/replicate. One-way ANOVA and Tukey’s multiple comparisons test. (**g**) Percentage of animals surviving to adulthood after larval induction of *FLP-out* somatic clones of a *Ras* transgene using *Tubulin-Gal4* (3 replicate experiments, N = 32–55 animals/genotype/replicate). One-way ANOVA and Tukey’s multiple comparisons test were used for statistical comparisons. (**h**) Images representing the leg imaginal disc *Tubulin-Gal4 FLP-out* clone sizes generated in the indicated genotype backgrounds. Scale bars = 20 um. (**i**) The mean +/- SEM leg imaginal disc clone size in pixels for each genotype (3 replicate experiments, N = 14–36 animals per replicate and N = 20–36 clones per replicate). One-way ANOVA and Tukey’s multiple comparisons test were used for statistical comparisons. ****p<0.0001. ***p<0.001. **p<0.01. n.s., not significant.

To identify Ras/MAPK molecular regulators that differentially impact strong or weak signaling outputs, we required a platform to tightly control the strength of MAPK signaling. To activate the pathway, we expressed a highly conserved, mutant active (G12V) *Drosophila Ras* transgene (termed *Ras*^*V12*^ here for convenience). To control MAPK signaling strength during fly development, we opted for the new approach of simply changing the codon usage of a *Ras*^*V12*^ transgene. Codons that occur infrequently in a given genome (rare codons) are known to impede translation, including in *Drosophila* [29–35]. By engineering a gene enriched in rare codons for each given amino acid, it is possible to create an mRNA that is poorly translated without altering the amino acid sequence of the encoded protein [36,37]. This has the distinct advantage that control of protein expression is embedded in the DNA and requires no additional factors or experimental variables. We used established data on *Drosophila* codon usage (see Methods) and created four distinct versions of *Drosophila Ras* transgenes: *1*) we altered none of the codons (*Ras*^*V12*^*Native*), *2*) we made all codons the most commonly occurring in the genome (*Ras*^*V12*^*Common*), *3*) we made all codons the most rare in the genome (*Ras*^*V12*^*Rare*), and *4*) we created a control wild-type version lacking the V12 mutation and also lacking codon alteration (*Ras*^*WT*^*Native*). To monitor expression, all four transgenes were epitope-tagged at the N-terminus with a 3XFLAG-tag sequence and expressed under the control of a Gal4-inducible UAS promoter (**Fig 1A**, see Methods). We note that *Ras*^*V12*^*Native* has primarily common codons and a similar Codon Adaptation Index (CAI [38]) to *Ras*^*V12*^*Common* [24], while the CAI for *Ras*^*V12*^*Rare* is much lower (**S1A and S1B Fig**). To control for position effects, all transgenes were integrated at the same site in the genome (see Methods). Our altering of the codon sequence yielded a *Drosophila Ras*^*V12*^*Rare* transgene that has a closer nucleic acid identity to the human KRASB isoform than the endogenous *Drosophila Ras85D* sequence (**S1C–S1E Fig**).

To measure signaling output strength of our transgenes, we first chose to use an *in vivo* phenotypic readout rather than a biochemical readout, an approach validated by quantitative studies of MAPK activation in *Drosophila* embryos [20,21]. For genetic screening of Ras/MAPK phenotypic regulators, the *Drosophila* eye is a highly accessible model. Driving expression of *Ras*^*V12*^ in the developing eye with an eye-specific promoter such as *sevenless* (*sev*) dysregulates the proper differentiation of the R7 photoreceptor cell, leading to an easily scored ‘rough-eye’ phenotype [39,40]. This phenotype relies on Ras action through the conserved MAPK pathway [2,41].

We assayed the phenotypic output of each *Ras* transgene *in vivo* by driving their expression in the developing fly eye using *sevenless (sev)-Gal4*. As expected [39], expression of *Ras*^*WT*^*Native* in this manner does not result in a rough-eye phenotype (**S2A Fig**). However, when we expressed the constitutive-active versions of *Ras (Ras*^*V12*^*)*, we found a range of rough-eye phenotypes **(Fig 1B)**. We binned these phenotypes into one of three classes: severe, moderate, or mild. Each class was assigned an increasing numeric score, based on the incidence and severity of eye phenotypes such as necrotic spots and discoloration (**Fig 1B,** see Methods). We then calculated an average severity score for each *Ras* transgene. *Ras*^*V12*^*Native* and *Ras*^*V12*^*Common* animals exhibit a similar phenotypic score, reflecting their similar CAI. Further, this phenotypic score is, on average, approximately 2-fold more severe than that of *Ras*^*V12*^*Rare* (**Fig 1C**). To determine whether Ras protein levels track with the difference in Ras-driven rough-eye phenotype, we isolated heads from flies encoding common and rare *Ras*^*V12*^ transgenes and performed serial dilution immunoblotting with an anti-FLAG antibody (**Figs 1D and S2D**). Separately, flies expressing all three active *Ras* transgenes were again immunoblotted with an anti-FLAG antibody, and protein levels were normalized to a loading control (**S2B and S2C Fig**). In both experiments, we found *Ras*^*V12*^*Common* flies express roughly 1.5 to 1.9-fold more Ras protein than flies expressing *Ras*^*V12*^*Rare* (**Figs 1D, and S2B–S2D**). Additionally, Ras^V12^ protein levels are similar between *Ras*^*V12*^*Native* and *Ras*^*V12*^*Common* flies (**S2B and S2C Fig**), which is consistent with the similar codon content between these transgenes (**S1 Fig**). These experiments established that codon usage can be manipulated to examine an *in vivo*, *Ras* signal-driven output (eye phenotype), and identified both weak (*Ras*^*V12*^*Rare*) and strong (*Ras*^*V12*^*Common*) versions of this output. Further, these results are consistent with *Ras*^*V12*^*Rare* serving as a model of the rare codon bias of human KRAS, the most commonly mutated RAS family member in human cancers.

We next assessed the impact of codon content in the *Ras* gene on *Ras* signaling and *Ras* GTPase activity. To examine the effect of expression of *Ras*^*V12*^*Rare* versus *Ras*^*V12*^*Common* transgenes on MAPK signaling, we measured the level of phosphorylated Mek (p-Mek, FlyBase: *Dsor*) and Erk (p-Erk, FlyBase: *rolled*) compared to the total level of these proteins by immunoblot analysis. *Ras*^*V12*^*Common* animals exhibit elevated levels of p-Erk and p-MEK compared to *Ras*^*V12*^*Rare* fly heads (**Figs 1E-** see figure legend for quantitation **and S2E**). We independently verified this difference in cultured S2 and KC insect cells (see Methods), again finding that *Ras*^*V12*^*Common* is expressed higher and more robustly activates the MAPK pathway compared to *Ras*^*V12*^*Rare* (**S1F Fig**). Further, using a Ras binding domain (RDB) pull-down assay [42], we found that S2 cells expressing *Ras*^*V12*^*Common* contain a higher total level of active Ras than cells expressing *Ras*^*V12*^*Rare* (**S1G Fig**). In sum, our findings establish *Ras*^*V12*^*Rare* and *Ras*^*V12*^*Common* as two distinct transgenes that either weakly or strongly activate Ras/MAPK signaling output (as measured by *Ras*^*V12*^ protein expression, Ras activity, and MAPK activation), and that transgene-driven signal strength tracks with an observable difference in phenotypic output.

Codon bias has been shown to impact not only translation fidelity and efficiency [43–47] but also pre-translational processes, including transcription [48,49] and mRNA stability [50–53]. To assess whether our codon-altered Ras transgenes impact Ras protein levels and Erk signaling through pre-translational processes, we performed quantitative RT-PCR (Methods). Paralleling our findings with Ras protein, *Ras*^*V12*^*Common* mRNA is significantly higher than *Ras*^*V12*^*Rare* in adult heads (**Fig 1F**). These findings are consistent with the model that altering codon usage of *Drosophila Ras* impacts *Ras* RNA, Ras protein, and Erk signaling.

We next examined the impact of *Ras*^*V12*^*Rare* and *Ras*^*V12*^*Common* at single cell resolution. Using the FLP-out system [54], we generated mosaic clones of cells throughout developing larvae that expressed these transgenes under a ubiquitous *Tubulin-Gal4* driver. Clones generated in this system are marked with GFP. We used heat shock to control the frequency of FLP-out events, and used a level of heat shock that resulted in 1–2 discrete clones in leg imaginal discs (see Methods). In these animals, *Ras*^*V12*^*Common*, but not *Ras*^*V12*^*Rare* or *Ras*^*WT*^*Native*, mosaic expression leads to animal lethality (**Fig 1G**). We note that *Ras*^*V12*^ expression is connected to animal lethality in other contexts, including when induced transiently or in somatic clones [55–57]. In surviving animals, *Ras*^*V12*^*Rare* significantly increases clone size relative to controls (**Fig 1H and 1I**), which is consistent with the well-known role of Ras/Erk signaling in promoting cell proliferation. Interestingly, in surviving *Ras*^*V12*^*Common* animals, clones are no bigger than in *RasNative* controls (**Fig 1H and 1I**). Taken together with the organismal death and frequent necrotic spots seen in the eyes of *sev-Gal4*, *UAS-Ras*^*V12*^*Common* animals, we interpret this result to likely reflect the increased apoptosis or cellular senescence that can result from increased Ras expression [58,59]. Given that *Ras*^*V12*^*Rare* and *Ras*^*V12*^*Common* have such differing effects on cell proliferation in leg discs, our results underscore the critical importance of signal output levels on *Ras-*driven phenotypes and highlight that lower Ras levels can actually drive more cell proliferation in specific contexts.

### A genome-wide screen uncovers differential phenotypic regulation between strong and weak Ras/MAPK signaling states

We next sought to use our codon alteration system to gain insight into how the Ras/MAPK pathway can be differentially regulated in different signal-strength states. To do so, we screened for molecular regulators that modify Ras/MAPK phenotypes driven only by strong or only by weak signaling states. We first confirmed that *Ras*^*V12*^*Common* and *Ras*^*V12*^*Rare* rough-eye phenotypes were both in the range that can be modified. Specifically, two different heterozygous loss-of-function mutations known to suppress active *Ras* phenotypes, namely the *S-627* allele of ***k****inase* ***s****uppressor of* ***r****as*, (FlyBase: *ksr*) [9], and the *S-2554* allele of ***b****eta subunit of type I* ***g****eranyl***g***eranyl* t*ransferase*, (FlyBase: *betaggt-I*) [3]. As with previous work, we find these mutations suppress the rough-eye phenotype for *Ras*^*V12*^*Common* and *Ras*^*V12*^*Rare* (**S3A Fig**). Next, we examined heterozygous mutants of the *yan-XE18* allele of ***a****nterior* ***op****en*, or *aop*, which is known to enhance the active Ras phenotype [60,61] Although we did not observe clear eye enhancement for *aop* ^*yan-XE18*^ */+*, we did observe a marked decrease in another phenotypic readout- animal survival. As for our FLP-out experiments with *Tubulin-Gal4*, *sev-Gal4* expression of *Ras*^*V12*^*Common* leads to considerably more organismal death than with *Ras*^*V12*^*Rare* (**S2B Fig**). This *sev-Gal4*-driven lethality likely reflects the expression of *sevenless-Gal4* in other tissues [62]. Survival is lower for *aop* ^*yan-XE18*^ */+* animals expressing both *Ras*^*V12*^*Common* and *Ras*^*V12*^*Rare* transgenes (**S3B Fig**). These results establish that codon-altered *Ras*^*V12*^ transgenes are subject to phenotypic modification, including by dose-sensitive heterozygous mutations.

**Fig 2:**
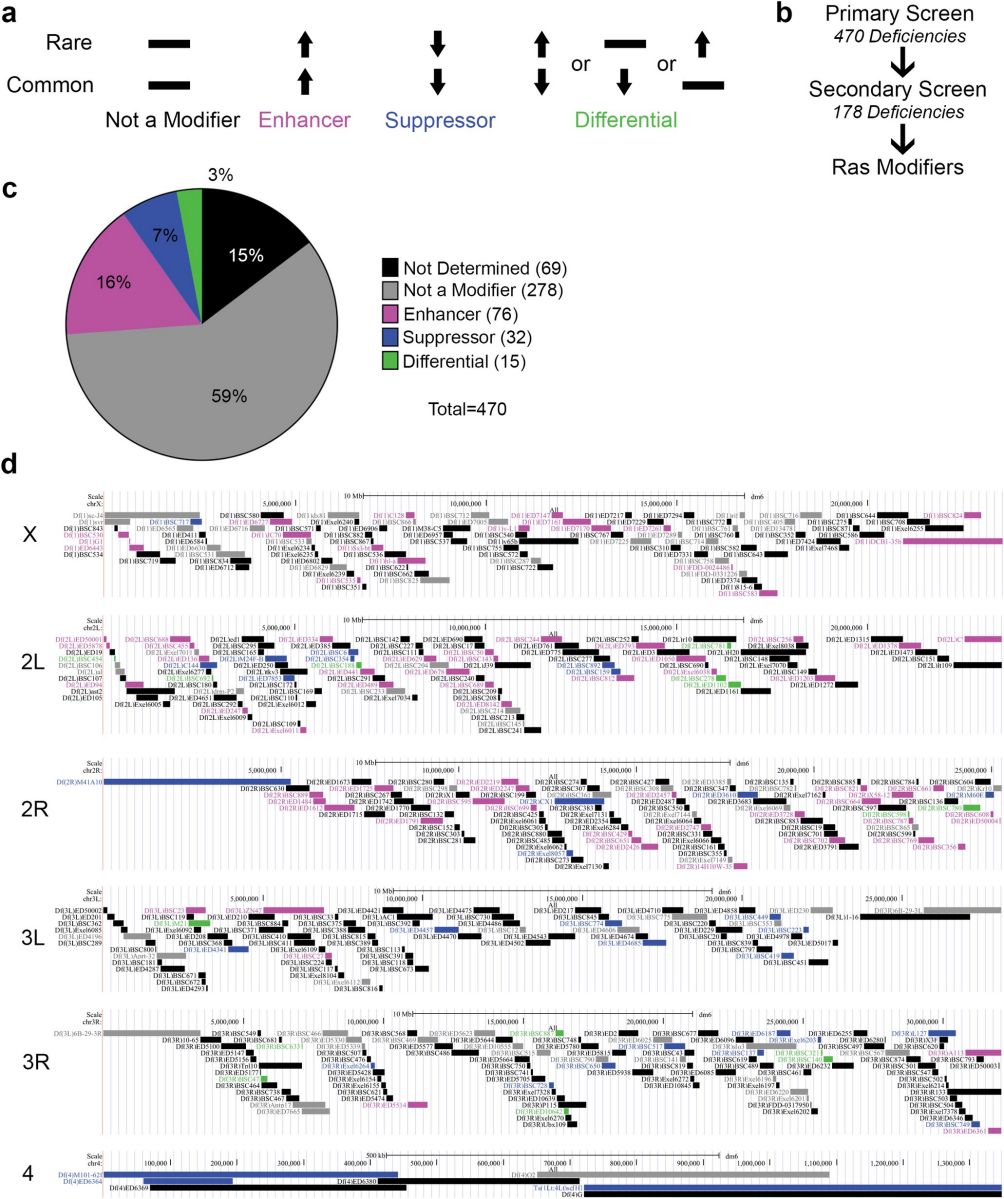
A genome-wide screen uncovers differential phenotypic regulation between strong and weak Ras/MAPK signaling states. **(a)** Schematic of the *Ras* modifiers types scored in the *Df* screen. **(b)** Schematic of screening approach. **(c)** Pie chart showing the number of *Df* with the indicated types of *Ras* modifiers. **(d)** Genome map of deficiencies color coded as in **c** for the class of *Ras* modifier.

Previous modifier screens, including in the eye, employed the native *Ras* cDNA to express activated *Ras* [2,8,40,63]. This sequence has a strong common-codon bias (**S1B Fig**) and is similar to *Ras*^*V12*^*Common* in terms of MAPK biochemical and phenotypic outputs **(Fig 1).** To find unidentified modifiers that may be specific to weaker (or stronger) Ras/MAPK-driven phenotypes, we conducted a genome-wide unbiased heterozygous mutant screen to specifically identify modifiers of the rough-eye phenotype driven by only *Ras*^*V12*^*Rare*, (or only *Ras*^*V12*^*Common*), (**Fig 2A**). We used the Bloomington Deficiency (*Df*) Kit, which covers 98.3% of the euchromatic genome^[^^64^^]^. In a primary screen (**Fig 2B and S1 Table**), we crossed 470 *Dfs* representing 99.1% of the *Df* collection to animals with *Ras*^*V12*^*Rare* or *Ras*^*V12*^*Common* expressed in the eye by *sev-Gal4*, and scored the resulting eye severity in an average of 30 (*Ras*^*V12*^*Common*) or 60 (*Ras*^*V12*^*Rare*) progeny animals per cross. We also factored animal lethality into our scoring (see Methods).

**Fig 3:**
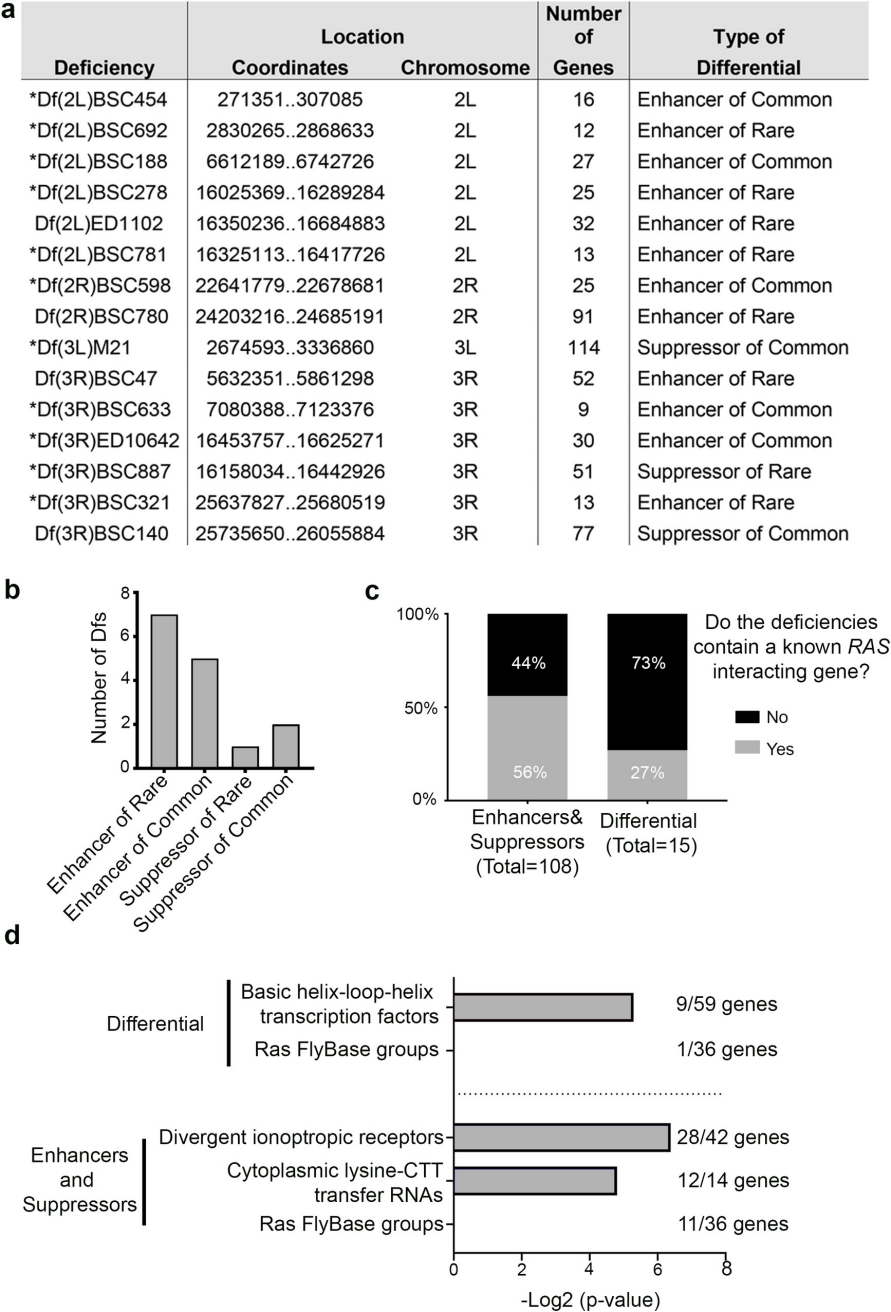
Characterization of differential modifiers. **(a)** Characterization of the differential *Ras* modifiers identified. Asterisks = those *Df*s for which no known *Ras* modifier has been reported (see Methods). **(b)** Pie chart showing the number of differential modifiers with the indicated phenotypes. **(c)** Graph of the percent (and number) of *Dfs* that do or do not contain known *Ras* interacting genes. **(d)** Enriched FlyBase gene groups contained in differential versus enhancer and suppressor deficiencies. The x-axis indicates the Log_2_ of the adjusted P value.

As expected, we found general *Ras* modifiers that either enhance or suppress eye phenotypes driven by both *Ras*^*V12*^ transgenes (**Fig 2C and 2D and S1 Table**). Interestingly, we identified more enhancers than suppressors (16% versus 7%, **Fig 2C**). The reason for this remains to be determined, but we note that our calculation of phenotypic modification (see Methods) included scoring animal lethality, which may identify strong enhancers of *Ras*^*V12*^*Common* not identified in previous screens based solely on a rough-eye phenotype. Of great interest, we also identified *Dfs* whereby *Ras*^*V12*^*Common* and *Ras*^*V12*^*Rare* are differentially modified (**Fig 2A**), meaning they scored as only modifying the eye phenotype driven by a single signaling state (*Ras*^*V12*^*Common* or *Ras*^*V12*^*Rare*, not both). Using a low-stringency cutoff score (see Methods), we identified 178 putative differential modifier *Dfs* in our primary screen (**Fig 2B and S1 Table**). To filter our hits to those that were the most robust, these *Dfs* were then re-tested in a secondary screen (**Fig 2B**) by crossing them a second time to *sev-Ras*^*V12*^*Common* and *sev-Ras*^*V12*^*Rare*. In this screen, we used a more stringent cutoff score to ensure repeatability to define a robust differential modifier (see Methods). This scoring and replicate analysis reduced the number of candidates to 15 *Dfs*, or 3% of the tested *Dfs* (**Fig 2E and 2D and S1 Table**), that reproducibly differentially modify either only *Ras*^*V12*^*Common* or only *Ras*^*V12*^*Rare* (**Fig 3A**). Among these differential modifiers, we again recovered more enhancers than suppressors, although importantly we recovered both enhancers of *Ras*^*V12*^*Common* and suppressors of *Ras*^*V12*^*Rare*, arguing that our screen had the dynamic range to modify both strong (*Ras*^*V12*^*Common*) and weak (*Ras*^*V12*^*Rare*) Ras/MAPK signaling outputs (**Fig 3B**).

We next queried both the general (signal output-independent) and differential (signal output-dependent) modifiers against a FlyBase database of all reported *Ras* genetic enhancers and suppressors (see Methods). 56% of our general modifier *Dfs* covered regions of the genome containing reported *Ras* enhancers or suppressors. These data support the idea that our approach can identify *Ras* eye modifiers. Additionally, we note that among our identified differential modifier *Dfs*, most (73%) do not encompass known *Ras* modifiers, supporting the idea that our signal strength-specific modifier hits are enriched in new *Ras* enhancers and suppressors (**Fig 3C**). To explore possible relationships amongst these 15 differential modifier *Dfs*, we queried the genes within differential versus enhancer and suppressor *Dfs* against the established list of FlyBase Gene Groups (FBGG). Interestingly, the gene groups enriched in the differential *Dfs* do not overlap with those in the general enhancer/suppressor *Dfs* (**Fig 3D**), suggesting that the differential modifiers may represent a distinct class of Ras modifiers. Unlike the general modifier Dfs, differential modifier regions are enriched for basic Helix Loop Helix (bHLH) transcription factors, potentially reinforcing their distinct regulation of Ras/MAPK signaling. In summary, by controlling Ras/MAPK signal output strength through codon usage and using a phenotypic output screen, we successfully identified *Dfs* that alter a Ras/MAPK phenotype in a signaling output-specific fashion.

### RpS21 negatively regulates Ras/MAPK signaling in a signal strength-specific manner

**Fig 4:**
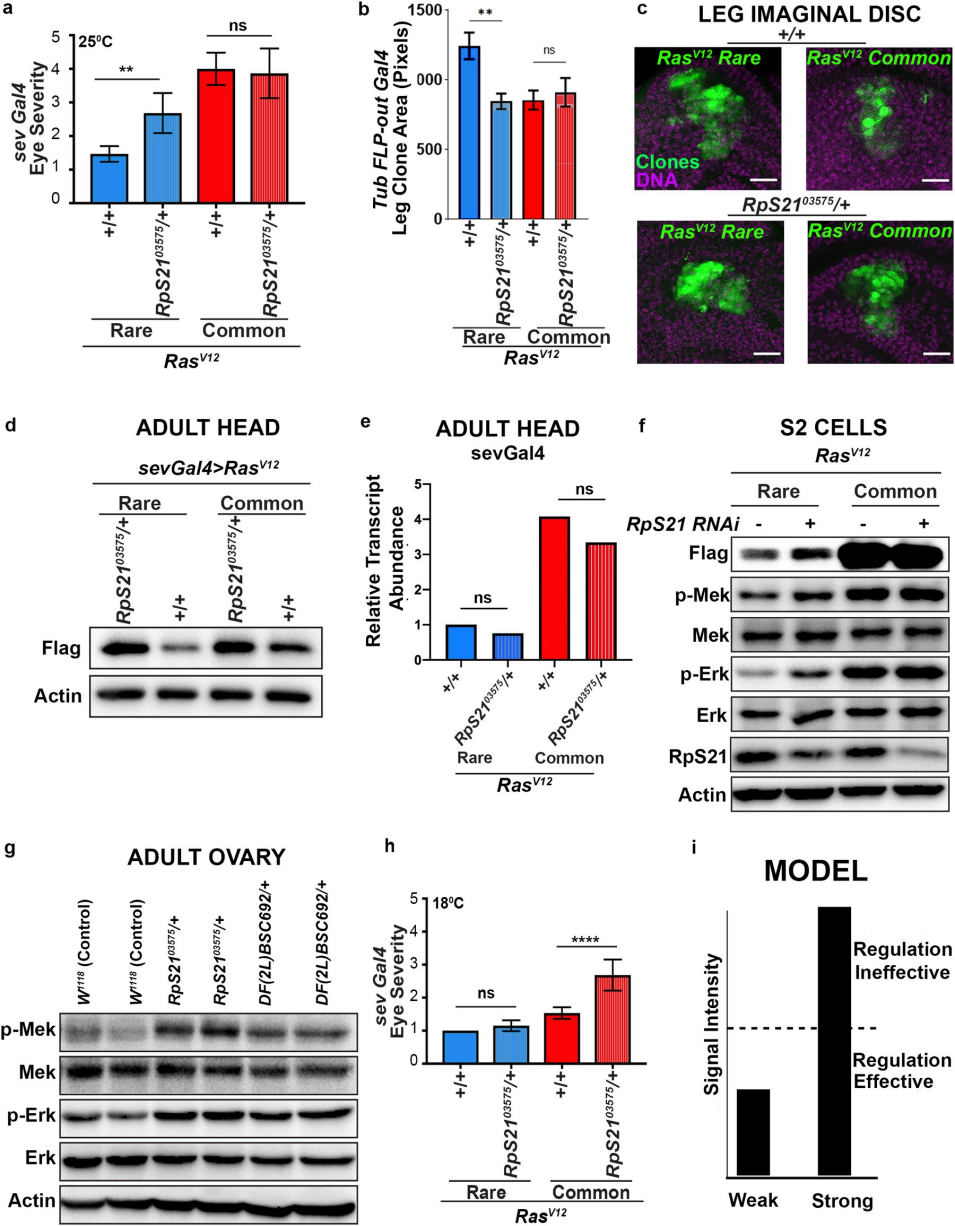
RpS21 negatively regulates Ras/MAPK signaling in a signal strength-specific manner. **(a)** The mean ± SEM eye severity score of the genotypes from three replicate experiments at 25°C. **(b)** The mean +/- SEM leg imaginal disc clone size in pixels for each genotype (3 replicate experiments, N = 10–33 total animals. One-way ANOVA test. **(c)** Images representing the leg imaginal disc *Tubulin-Gal4 FLP-out* clone sizes generated in the indicated genotype backgrounds. Scale bars = 20 um. (**d**) Immunoblot detection of transgenic Ras^V12^ (with an anti-FLAG antibody), and actin as a loading control from lysates derived from the heads of flies with the indicated versions of transgenic *Ras*^*V12*^ in either the wild-type (+/+) or mutant (*RpS21*^*03575*^*/+)* RpS21 backgrounds. **(e)** Quantitative RT-PCR, measured using 2^ΔΔCt, of animals expressing the indicated versions of transgenic *Ras*^*V12*^ in either the wild-type (+/+) or mutant (*RpS21*^*03575*^*/+) RpS21* backgrounds. Data represent three independent replicates per condition, with 10–40 animals/replicate. One-way ANOVA and Tukey’s multiple comparisons test. **(f)** Immunoblot detection of transgenic Ras^V12^ (with an anti-FLAG antibody), phosphorylated (p-) and total Mek and/or Erk, RpS21, and actin as a loading control from lysates derived from S2 cells stably transduced with expression vectors expressing the indicated *Ras*^*V12*^ transgenes in the absence (-) and presence (+) of *RpS21* RNAi. **(g)** Immunoblot detection of indicated proteins derived from lysates of the adult ovaries of either wild-type (*+/+*) or mutant (*RpS21*^*03575*^*/+*) flies. **(h)** The mean ± SEM eye severity score of the genotypes from three replicate experiments at 18°C. Tukey’s multiple comparisons test was used for statistical comparisons in **a** and **e**. ****p<0.0001. **p<0.01. n.s., not significant. **(i)** Model depicting how a signal intensity modifier such as RpS21 may be ineffective above a specific signaling intensity threshold (dotted line).

To identify a differential modifier from our screen at the single gene level, we focused on *Df(2L)*BSC692 as it was one of the smallest deficiencies, encompassing only 12 genes, that specifically enhanced *Ras*^*V12*^*Rare* (**Figs 3A and S4A**). Of these 12 genes, ***R****ibosomal* ***p****rotein* **S21**, or RpS21 (also known as *overgrown hematopoietic organs 23B/oho23B)*, represented a plausible candidate modifier. RpS21 stands out among small ribosomal subunits for its reported negative regulation of hematopoietic and imaginal disc hyperplasia [65]. To determine if *RpS21* is a responsible gene in *Df(2L)BSC692* for specifically enhancing *Ras*^*V12*^*Rare*, we assessed the rough-eye phenotype of *Ras*^*V12*^*Common* and *Ras*^*V12*^*Rare* in the background of the mutant *RpS21*^*03575*^. Indeed, only the *sev-Ras*^*V12*^*Rare* rough-eye phenotype is enhanced in the *RpS21*^*03575*^*/+* background (**Fig 4A**). *RpS21*^*03575*^*/+* did not score as a hit by our animal lethality criteria (see Methods), suggesting our comparison of eye phenotypes between control and mutant animals was not impacted by animal viability. We also note that the *RpS21*^*03575*^ chromosome also carries a mutation in *cinnabar (cn)*. However, *cn* mutations were also present in 4 other Dfs in our screen, only one of which was a hit. Therefore, *RpS21* and not *cn* is the likely modifier on the *RpS21*^*03575*^ mutant chromosome. Similar to our findings in the eye, *RpS21*^*03575*^*/+* preferentially impacts the phenotype of *Ras*^*V12*^ *rare* leg imaginal disc clones. We observe smaller average clone sizes in RpS*21*^*03575*^*/+*, *Ras*^*V12*^*Rare* animals relative to *Ras*^*V12*^*Rare* alone, whereas *RpS21*^*03575*^*/+* does not impact clone size in *Ras*^*V12*^*Common* animals (**Fig 4B and 4C**). Together, these findings identify *RpS21* as a responsible modifier of *Ras*^*V12*^*Rare* in one *Df* from our Ras/MAPK signal strength-specific screen.

From our genome-wide screen and follow-up mapping efforts, we were able to identify both an *RpS21* mutant allele and a small deficiency encompassing this gene (*Df(2L)BSC692)* as differential *Ras*^*V12*^ eye phenotype modifiers. We next examined the molecular alterations of Ras signaling that underlie this signal intensity-specific modification. To this end, we assessed Ras^V12^ levels and/or MAPK pathway activation by immunoblot analysis in three distinct cellular and signal output settings: ectopic Ras activation in adult fly heads, ectopic Ras activation in cultured S2 cells, and endogenous MAPK signaling in ovaries. Our results overall show that while RpS21 reduction impacts Ras/MAPK in numerous settings, there is a more pronounced effect in cases where signaling output is weaker.

In the heads of *Ras*^*V12*^*Rare* flies, transgenic Ras protein levels increase in *RpS21*^*03575*^*/+* animals relative to wild type. This result is consistent with the enhanced *Ras*^*V12*^*Rare* eye phenotype in RpS*21*^*03575*^*/+* animals. However, unlike our lack of an observable phenotypic enhancement of *Ras*^*V12*^*Common* in the eye, at the biochemical output level we also observe an increase in the level of Ras^V12^Common in the *RpS21*^*03575*^*/+* background (**Figs 4D and S4B**). This result shows that *RpS21*^*03575*^ modifies both *sevenless-*driven Ras^V12^Rare and Ras^V12^Common protein levels in the adult fly head, but only Ras^V12^Rare modification leads to an observable phenotypic output in this setting. This difference between eye phenotype and protein level effects could suggest that a large difference in Ras protein change is needed to cause a detectable change at the eye phenotype level. Alternatively, our adult head assay focuses on Ras levels in the adult animal, whereas our eye assay focuses on the effect of RpS21 reduction during eye development. *RpS21*^*03575*^*/+* does not impact *Ras*^*V12*^*Rare* or *Ras*^*V12*^*Common* RNA levels in adult heads, suggesting *RpS21* acts at the translational level to impact Ras signaling (**Fig 4E**).

Next, we examined the impact of RpS21 on Ras signaling in additional cellular contexts. We first transduced S2 cells with an expression vector encoding either *Ras*^*V12*^*Common* or *Ras*^*V12*^*Rare*, and then used RNAi to reduce RpS21 levels. As in the fly head, *RpS21*^*RNAi*^ elevates Ras^V12^Rare protein levels. However, unlike in the head, Ras^V12^Common protein levels in S2 cells are unaffected by *RpS21*^*RNAi*^ (**Figs 4F and S4C**). We note that, in these cells, our expression system led to particularly robust expression of the Ras^V12^Common protein (**Figs 4F and S4C**). We also examined MAPK activation in S2 cells. Whereas p-Mek and p-Erk are noticeably increased in *RpS21*^*RNAi*^ S2 cells expressing *Ras*^*V12*^*Rare*, we see no overt increase in these MAPK activation readouts upon *RpS21*^*RNAi*^ in S2 cells expressing *Ras*^*V12*^*Common* (**Figs 4F and S4C**). Taken together, our results in the head and in S2 cells suggest that when Ras signaling is above a particular threshold (e.g., *Ras*^*V12*^*Common* expression in S2 cells), RpS21 reduction does not impact pathway output.

We also assessed whether endogenous MAPK signaling can be regulated by RpS21 *in vivo*. To do so, we examined the effect of disrupting one allele of the *RpS21* gene on endogenous MAPK signaling in the ovaries of flies, a tissue where EGFR/Erk signaling has a well-defined role [66,67] and where phosphorylated Mek and Erk are readily detected (**Fig 4G**). Of note, *RpS21*^*03575*^*/+* animals have no obvious female fertility defects. In this tissue, endogenous p-Mek and p-Erk levels increase in both *Df(2L)*BSC692/+ and *RpS21*^*03575*^*/+* animals relative to control *w*^*1118*^ animals (**Fig 4G**). Although we were not able to successfully determine endogenous Ras levels in the ovary with existing reagents (not shown), our overall findings are consistent with RpS21 negatively regulating endogenous Ras/MAPK signaling in this tissue. Collectively, we find that loss of RpS21 elevates Ras/MAPK signaling in multiple contexts.

Our immunoblot analysis validates our genetic screen finding that RpS21 can negatively regulate Ras and/or MAPK signaling, in a manner that potentially depends on the strength of Ras/MAPK signaling. One interpretation of these data is that RpS21 has a minimal effect on MAPK signaling output above a certain threshold of MAPK signaling. Such a model would predict that experimentally reducing the amount of *Ras*^*V12*^*Common* expression should render fly eye development sensitive to the *RpS21*^*03575*^*/+* mutant background. To experimentally test this threshold model, we took advantage of the well-known fact that expression of transgenes using the Gal4-UAS system is responsive to temperature, with higher temperature resulting in higher expression over the physiological range of 18°C-29°C. We thus evaluated the rough-eye phenotype of *sev-Ras*^*V12*^*Common* versus *sev-Ras*^*V12*^*Rare* flies in a wild-type versus *RpS21*^*03575*^*/+* mutant background, only this time at 18°C. At this lower temperature, *RpS21*^*03575*^*/+* now acts as an enhancer of *Ras*^*V12*^*Common* (**Fig 4H**). Interestingly, *RpS21*^*03575*^*/+* no longer enhances *Ras*^*V12*^*Rare*, underscoring the sensitivity of *RpS21/+* to Ras/MAPK signaling strength. Therefore, RpS21 regulation of the Ras pathway appears to be signal-strength dependent, rather than codon-dependent. Collectively, these results demonstrate that while RpS21 negatively regulates Ras-MAPK signaling in diverse contexts, at the phenotypic level this regulation preferentially impacts weak Ras/MAPK signaling. These findings are consistent with a model whereby above a certain signaling intensity threshold, regulators that impact Ras signaling at weaker intensity levels are no longer effective (**Fig 4I**).

### RpS21 downregulation does not alter expression of a codon-altered GFP reporter

**Fig 5:**
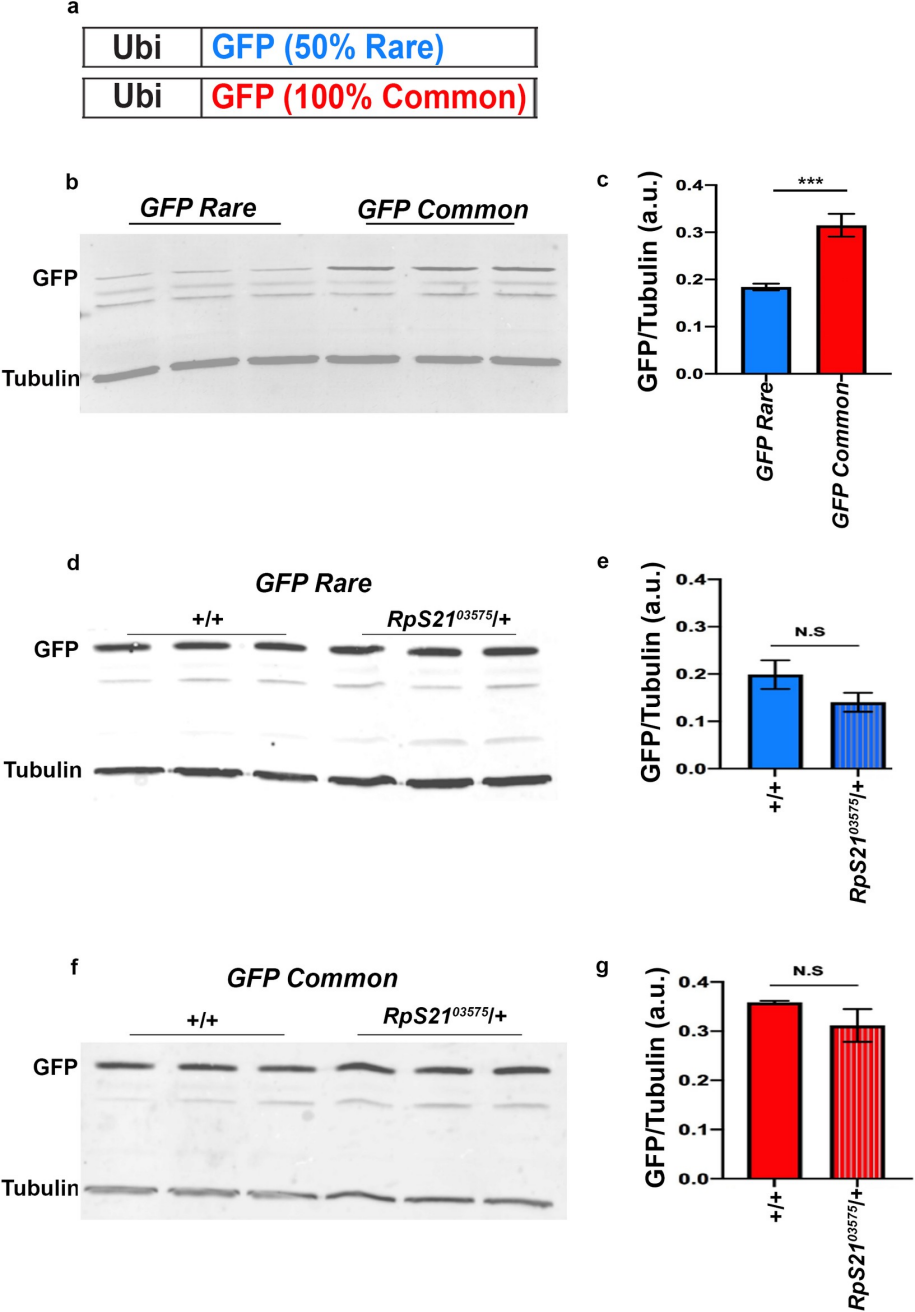
RpS21 downregulation does not impact codon-dependent GFP protein expression. **(a)** Schematic representation of the *GFP* transgenes encoded by rare or common codons. (**b**) Immunoblot detection of GFP protein and αTubulin as a loading control from lysates derived from adult flies with the indicated versions of transgenic *GFP*. (**c**) Quantification of protein levels for blot in **b**. a.u. = arbitrary units. Data represent mean ± SEM, 3 replicates, One-way ANOVA and Tukey’s multiple comparisons test. (**d,f**) Immunoblot detection of GFP protein and αTubulin as a loading control from lysates derived from adult flies with the indicated versions of transgenic *GFP*, and in the indicated genetic backgrounds. (**e,g**) Quantification of protein levels for blot in **d, f** respectively. a.u. = arbitrary units. Data represent mean ± SEM, 3 replicates, One-way ANOVA and Tukey’s multiple comparisons test. ***(p<0.001), N.S. = not significant.

Our above results suggested that it is Ras/MAPK signaling strength, and not codon manipulation specifically, that determine whether RpS21 heterozygosity impacts protein expression. To test this idea further, we generated an additional pair of transgenes with identical protein sequence but distinct codon usage. Specifically, we generated two GFP transgenes- one with GFP containing 100% common codons, and one where the same GFP had 50% synonymous substitutions of rare codons dispersed throughout the protein. Both transgenes were expressed under a *ubiquitin* promoter and were integrated into the same site in the genome (**Figs 5A and S5,** see Methods). Consistent with our results for altering codon content of the Ras gene, GFPCommon protein is expressed at a higher level in adult animals than GFPRare protein (**Fig 5B and 5C**). Given this, we next tested whether RpS21 downregulation alters GFP protein expression in a codon-dependent manner. *RpS21*^*03575*^*/+* animals exhibit similar GFPRare protein expression as wild type animals (**Fig 5D and 5E**). Additionally, *RpS21*^*03575*^*/+* animals exhibit similar GFPCommon protein expression as wild type animals (**Fig 5F and 5G**). These results indicate that RpS21 downregulation does not impact translation of at least one other tested transgene pair, suggesting that RpS21 may, to some degree, act specifically to regulate the Ras/MAPK pathway at specific signaling intensity levels. Overall, our findings highlight the ability of our approach to reveal new Ras/MAPK regulators that preferentially impact specific signaling outputs.

## Discussion

Here, we revisit a well-proven strategy to identify Ras/MAPK modifiers (a heterozygous mutant screen in the *Drosophila* eye) but do so with the new angle of altering codon usage in a core signaling component to find signal strength-dependent regulators. We show here that changing codon usage in a signaling pathway component can be an effective strategy to find signal strength-dependent modifiers, as evidenced by our identification of 15 *Df* from a whole-genome screen that only modify the rough-eye phenotype driven by either a common or rare codon-enriched *Ras*^*V12*^ transgene, but not both. From these efforts, we identify the *RpS21* gene as a negative regulator of a weak or low-level *Ras* phenotype in the *in vivo* context of eye development. These findings are further supported by our finding that RpS21 reduction in other contexts also impacts (low) endogenous Ras signaling in the ovary, but not higher Ras signaling in S2 cells.

Our results show that altering codon usage can serve as a valuable platform to stably alter protein production to undertake signal strength-specific screens. Clearly, there are other ways that one can modulate signal output strength, such as modulating gene expression strength as we also do here, or through use of an allelic series [68]. However, an advantage of altered codon usage is that it can be hard-wired into the genome, and thus no additional (and potentially confounding) experimental parameters such as altering temperature, inducing genes with drugs, and so forth are required. Our approach should be applicable to any signal transduction pathway. The utility of our approach is underscored in the fact that signal strength-specific modifiers found in our screen appear to be enriched for genome regions not previously linked to *Ras* genetic modification. The causative genes contained within 14 of these differential *Df* hits remain to be mapped and represent a potentially rich source of new genes modulating Ras/MAPK signaling. Previous work found that different levels of MAPK activity impact different biological processes [68]. Intriguingly, our differential hits appear to be enriched in bHLH transcription factors. Of note, the bHLH transcription factor Myc is a well-known Erk target [69–72], and it will be interesting to explore whether specific bHLH transcription factors are preferentially targeted by this pathway in signal strength-dependent contexts.

Given the importance of Ras/MAPK signaling in many settings across evolution, our identified modifiers may shed insight into how this pathway is controlled at different signal strengths. While our focus here is on *Drosophila* eye development, signal strength dependencies of the Ras/MAPK pathway are appreciated to play a role in human disease. Activating mutations in the MAPK pathway of humans underlie a class of human diseases termed RASopathies [73]. Further, relevant to our approach here, of the three human RAS genes, *KRAS*, is the most enriched in rare codons [27] and is the most commonly mutated RAS isoform in human cancers [28,74]. Changing the rare codons to more common codons in a single exon of the mouse *KRAS* gene leads to fewer tumors following carcinogen exposure [22], which is in line with current thinking on a “sweet spot” level of Ras/MAPK signaling required to initiate tumorigenesis [28]. We argue that the larger clone size that we observe in leg imaginal discs of animals expressing *Ras*^*V12*^*Rare* vs. *Ras*^*V12*^*Common* reflects this same concept. As such, the new tools we report here may provide valuable reagents to more accurately model KRAS-relevant regulation in *Drosophila* and ultimately in KRAS-driven disease.

Our approach found that RpS21 functions as a negative regulator of weak Ras/MAPK signaling. While one might expect that a codon-based approach would pull out ribosomes as hits, we show here that codon-independent manipulation of Ras signaling, through temperature change, confirms that RpS21 is responding to specific signaling levels rather than specific codons. As Ras/MAPK signaling is known to drive tissue growth in diverse settings, this may suggest that RpS21 can function as a negative regulator of tissue or tumor growth. Interestingly, downregulation of RpS21 was previously shown to cause excessive hyperplasia in hematopoietic organs and imaginal disc overgrowth during larval development, suggesting RpS21 acts as tumor suppressor in *Drosophila* [65]. Although this finding may seem paradoxical given that ribosomal mutants in flies are well-known to cause minute phenotypes, characterized by short bristles, small body size, and delayed growth [75–78], a subset of ribosomal proteins including RpS21 have been identified to have a growth suppressive role [65,79–83]. Further, heterozygosity of many ribosomal proteins is reported to be tumorigenic in zebrafish [84], and heterozygous inactivating mutations of ribosomal proteins have been described in human cancers [85,86]. Several mechanisms have been proposed to account for this apparent tumor suppressor activity of ribosome protein downregulation, including activation of p53 [87–89], inhibition of NF-KB [90], E2F [91], MYC [92], and CDK8 [93]. Thus, RpS21 joins the ranks of an emerging number of ribosomal proteins with roles in growth suppression, although whether RpS21 acts as a tumor suppressor in mammals awaits investigation.

The mechanism underlying the negative regulation of Ras/MAPK signaling by RpS21 remains to be determined. Future work can explore how direct the regulation is, and whether RpS21 acts in a cell autonomous or, has been shown for a subset ribosomal subunits, a non-autonomous manner to regulate tissue growth [83]. Future work can also explore whether other signaling pathways connected to eye development are also impacted by RpS21 reduction. In our work, we found that RpS21 downregulation promotes elevated levels of Ras^V12^ protein in multiple settings. The effect of RpS21 on Ras^V12^ protein level could potentially be through RpS21’s canonical ribosomal function or through an extra-ribosomal function. Dose-dependent ribosome dysfunction is linked to the human disease Diamond-Blackfan anemia, where heterozygous mutations in specific ribosomal subunits are linked, at least in part, to compromised ribosome biogenesis and translation [94–97]. A defect in RpS21 ribosomal function may trigger ribosomal biogenesis defects that alter translational fidelity or promote generation of oncoribosomes to preferentially express subset of mRNA pools [98,99]. Alternatively, RpS21 might participate in other cellular processes independent of its canonical ribosomal function, as has been shown for other ribosomal subunits [100–103].

We note that RpS21 has been connected to positive regulation of Ras/MAPK in other contexts. While this manuscript was in review, a recent study revealed that downregulation of human RPS21 inhibits metastatic behavior of osteosarcoma cells in a MAPK-dependent manner [104], underscoring the potential human relevance of our findings here. Further, in contrast to our screen results revealing negative regulation by RpS21 in multiple contexts, numerous ribosomal proteins (RpS21 included) were found among 1,162 genes to positively regulate Erk phosphorylation in a previous primary screen in cultured *Drosophila* S2R+ cells [15]. Unlike this Erk activation screen, we note that our *Ras*^*V12*^ eye modifier screen hits were not preferentially enriched for ribosomal subunits, and that ribosomes in general are not enriched among known FlyBase Ras genetic enhancers/suppressors. We hypothesize that the addition of insulin to the growth media, required for Erk activation in the context of the S2R+ cell screen, revealed a dependency for cell growth, which is dependent on both ribosomes and Erk activation. S2R+ cells have known differences from S2 cells in response to external signaling, and this could reflect differences in MAPK regulation in this context as well [105], underscoring the need to understand signaling dynamics and regulation in a given biological context.

Another question for future investigation is why RpS21 regulation of Ras signaling is non-functional in contexts of heightened Ras/MAPK signaling, as we observed in S2 cells with strong Ras/MAPK biochemical output, as well as at the phenotypic output level where *Rps21/+* failed to noticeably modify the eye phenotype of *Ras*^*V12*^*Common*. One possible explanation is that different MAPK signaling strengths activate a different host of MAPK targets, and this impacts the degree of negative regulation by RpS21. To that end, it will be important to further mine our screen to identify single gene modifiers in the other 14 *Dfs*, which may similarly yield new regulatory insight into the Ras/MAPK pathway.

In summary, we show here the value of manipulating codon usage of one component of a pathway to modulate the corresponding signaling output, and the use thereof to screen for modifiers of specific signaling intensities. This approach proved successful, identifying a novel regulator of the Ras/MAPK pathway, RpS21. As such, this approach may find value in similarly interrogating other signaling pathways.

## Methods

### Generation of codon-altered genes in Drosophila

Codon-altered exon sequences for *Ras*^*V12*^*Common*, *Ras*^*V12*^*Rare*, *GFPRare*, and *GFPCommon* were created using the Kazusa codon usage database (https://www.kazusa.or.jp/codon/) and subsequently generated by Gene Synthesis (ThermoFisher Scientific, Invitrogen GeneArt). A cDNA clone (LD17536, Drosophila Genomics Resource Center) was used as a template to generate the non-altered *Ras85D* sequence. To generate *Ras*^*V12*^*Native*, the QuikChange II Site-Directed Mutagenesis Kit (Agilent) was used to change codon 12 in *Ras85D* from GGA (glycine) to GTA (valine). Subsequently, primers (sequences available upon request) were designed to amplify *Ras* sequences and the Invitrogen Gateway BP Clonase II Enzyme Mix (ThermoFisher Scientific) was used to insert these sequences into the Gateway entry vector pDONOR221 (ThermoFisher Scientific). Subsequently, the Invitrogen LR Clonase Enzyme Mix (ThermoFisher Scientific) was used to insert the *Ras*^*WT*^, and *Ras*^*V12*^ Native, Common, and Rare sequences into the Gateway destination vector pBID-UASC-FG (Addgene Plasmid #3520 [106]), which has a N-terminal FLAG tag and a PhiC31 site for site-directed genomic insertion. pBID-UASC-FG-*Ras* plasmids were prepared with a ZymoPURE II Plasmid Midiprep Kit (Zymo Research) and sent to Model System Injections (Durham, NC, USA) for injection into *attP40 (2L)* flies. *GFP* sequences were cloned into a pBID plasmid (modified from Addgene Plasmid #3520), and DNA and transgenic flies were prepared as for Ras transgenes. For cell culture, *Ras*^*V12*^*Common* and *Ras*^*V12*^*Rare* transgenes were cloned into pMKInt-Hyg vectors, which were sequenced to confirm the correct sequence.

#### Fly stocks

All flies were raised at 25°C on standard media unless noted otherwise (Archon Scientific, Durham NC). FlyBase (http://FlyBase.org) describes full genotypes for all stocks used in this study. See **S1 Table** for Df stock information. All other stocks were the following genotypes (Bloomington Drosophila Stock Center numbers in parentheses when available): *ksr*^*S-627*^*/TM3*,*Sb* (#5683), *aop*^*yan-XE18*^*/CyO* (#8777), *betaggt-I*^*S-2554*^ (#5681), *RpS21*^*03575*^*/CyO* (#11339), *hsflp;; UAS-GFP*, *tubulin-FRT-STOP-FRT-Gal4* [54], and the Bloomington Deficiency(Df) kit. The following stocks were generated for this study: *UAS-FLAG-Ras*, *UAS-FLAG-Ras*^*V12*^*Native*, *UAS-FLAG-Ras*^*V12*^*Common*, and *UAS-FLAG-Ras*^*V12*^*Rare*, *ubi-GFP Rare*, *ubi-GFP Common*.

#### Fly genetics and deficiency screen

To examine mitotic clones in leg imaginal discs and associated animal survival in such experiments, flies containing UAS *Ras* transgenes were crossed to *hsflp;; UAS-GFP*, *tubulin-FRT-STOP-FRT-Gal4* animals. F1 larvae were collected 96 hours after egg laying and heat shocked at 37 degrees for 20 minutes. After 24 hours, leg imaginal discs were dissected from living larvae. Discs were fixed as done previously for imaginal discs [107] and probed with DAPI for DNA. Images were taken on a Nikon A1 confocal microscope. Clone sizes were determined using FIJI’s Tracing and measuring tools.

To examine eye phenotypes and associated animal survival in such experiments, the *Ras* transgenes were combined with a *sev* Gal4 driver and subsequently crossed to *Df*/Balancer flies. After 16–18 days after egg laying, the rough eye phenotype of the resulting progeny was scored (both males and females). The scoring system was as follows (category = numerical score, qualitative description): Mild = 1, no discoloration or necrotic tissue; Moderate = 3, discoloration and no necrotic tissue; Severe = 5, discoloration and necrotic tissue (see **Fig 1B and 1C**). Severity scores for each genotype was calculated as follows: (#Mildx1+#Moderatex3+#Severex5)/Total # of flies. To determine if heterozygosity for a subset of genes altered the rough eye phenotype the following two genotypes for each deficiency (*Df*) were compared: *Ras* transgene only and *Ras* transgene + *Df* (used as an internal comparison to control for background effects). Then, we calculated a fold change score for both *Ras*^*V12*^*Common* and *Ras*^*V12*^*Rare* for each deficiency: *Ras* transgene + deficiency/*Ras* transgene. We note that none of the Df animals on their own had detectable eye phenotypes. For the primary screen, the fold change score was defined as follows: enhancer (fold change ≥1.35 or 5X less flies eclosed); suppressor (fold change ≤0.65 or 5X more flies eclosed). For the secondary screen, the fold change score was defined as follows: enhancer (fold change ≥1.95 or 5X more flies eclosed); suppressor (fold change ≤0.50 or 5X less flies eclosed). The final phenotype for a deficiency was defined as follows: not a modifier (neither *Ras*^*V12*^*Common* or *Ras*^*V12*^*Rare + Df* were modified); enhancer (both *Ras*^*V12*^*Common* and *Ras*^*V12*^*Rare + Df* were enhanced); suppressor (both *Ras*^*V12*^*Common* and *Ras*^*V12*^*Rare + Df* were enhanced); differential (only *Ras*^*V12*^*Common* or *Ras*^*V12*^*Rare + Df* were modified). We note that overall eye size was relatively unaffected by different Ras transgenes. Images of fly eyes were obtained using a Leica MZ10F microscope with a PlanApo 1.6X objective, Pixel Shift Camera DMC6200, and LASX software.

#### Protein preparation and analysis

All protein samples were prepared by homogenizing tissue on ice. For **Figs 5, S1B and S5** samples were processed in Laemmli buffer and then boiled for 5min. Samples were separated by 12% sodium dodecyl sulfate-polyacrylamide electrophoresis (SDS-PAGE) gels and transferred to an Odyssey nitrocellulose membrane (LI-COR Biosciences) for immunoblotting. The following antibodies were used: anti-FLAG M2 (1:500, Sigma, anti-mouse), anti-α-tubulin (1:20,000, Sigma, anti-mouse), rabbit anti-GFP (Life Technologies, #A11122), IRDye 800CW (1:20,000, LI-COR Biosciences, anti-mouse or anti-donkey), and Alexa Fluor 680 goat anti-mouse IgG (H+L) (Invitrogen, #A21058). Signal was detected using LI-COR Odyssey CLx and analyzed using Image Studio (LI-COR Biosciences). For all other immunoblots, samples were processed in RIPA buffer containing 1% IGEPAL, 50mM NaCl, 2mM EDTA, 100mM Tris-HCl, pH 8.0, 0.1% Glycerol, 50 mM Naf, 10mM Na3VO4, and protease inhibitors (Roche). *Drosophila* heads and ovaries were collected and transferred to cold lyses to be homogenized with a pellet pestle. Lysates were incubated at 4°C for 30 min on end-to-end rotator and then centrifuged at 21,000 x g for 10 min. The supernatant was transferred to a new tube. Total protein was quantified using a BCA kit (Bio-Rad) and 10 to 50 micrograms of protein was used for separation on either 12.5% or 15% gradient SDS-PAGE gels. Proteins on SDS gels were transferred onto polyvinylidene difluoride membranes. These membranes were probed with anti-Flag (Sigma, anti-mouse 1:1000), anti-β-actin (Cell Signaling, 1:1000), anti-p-MEK1/2 (Cell Signaling, 1:500), anti-MEK1/2 (Cell Signaling, 1;500), anti-p-ERK1/2 (Cell Signaling, 1:1000), anti-ERK1/2 (Cell Signaling, 1:1000), and anti-RpS21 (Abcam, 1:2000) primary antibodies in blocking buffer containing 5% milk goat anti-mouse IgG (H+L) HRP (Life Technologies, 1:10000) or goat anti-rabbit IgG (H+L) HRP (Thermo Fisher Scientific, 1:10000). Immunoblots were visualized using Clarity Max ECL Western Blotting Detection Reagent (Bio-Rad) followed by exposure to digital acquisition using Chemi Doc Imager (Bio-Rad). For all blots, the contrast and/or brightness were altered equally across the entire image and then images were cropped for displaying as figures. Flag band intensity was measured using Image Lab v6.0.1 software and then each band was normalized to the lowest intensity band. The active Ras detection kit (Cell Signaling, #8821) was used to detect GTP-bound Ras^V12^, both rare and common.

#### RT-PCR

Animals were aged 3–7 days at 25°C on standard fly medium. RNA was extracted from adult fly heads using TRIzol™ reagent (ThermoFisher, cat#15596026) according to the manufacturer’s protocol (10–40 heads per sample in 500ul TRIzol™ reagent). Purified RNA was resuspended in molecular grade water. RNA was DNase treated with DNase I at room temp for 15 minutes, then the reaction was terminated by adding 25mM EDTA and incubating at 65°C for 10 minutes. DNase efficiency was confirmed using a positive control. DNase treated RNA was reverse transcribed into cDNA using iScript cDNA synthesis kit (BIO-RAD, cat#170–8891) according to the manufacturer’s protocol. Subsequent cDNA was treated with RNase H prior to use in qPCR reactions. The concentration of the RNA was quantified on a NanoDrop spectrophotometer and samples were diluted with molecular grade water to match the concentration of the lowest concentration sample. Luna Universal qPCR Master Mix (NEB #M3003) was used to run the qPCR reaction according to the manufacturer’s specifications. Primers for the detection of *Ras* constructs were designed against an identical region containing the 3xFLAG sequence shared by both *Ras*^*V12*^*Common* and *Ras*^*V12*^*Rare* transcripts. Primers were designed against *Drosophila Beta Tubulin 56D* as a reference gene. Ras qPCR FW primer: TGGACTACAAAGACCATGACGGT, Ras qPCR RV primer: ACTTGTATACCGGTGCTTGTCAT, Tubulin qPCR FW primer: GGACGAGACCTACTGCATCG, Tubulin qPCR RV primer: GGTCACCGTATGTGGGTGTC.

#### Cell culture

KC and S2 cell lines were obtained from Bloomington (Indiana University DGRC Bloomington) and as a gift from Dr. David MacAlpine (Duke University) respectively. These cells were cultured in Schneider’s *Drosophila* medium (Invitrogen) supplemented with 10% fetal bovine serum (FBS) and 1% penicillin–streptomycin- L Glutamine (Invitrogen) at 25°C. FBS was heated for 60 minutes in 58°C and then cooled down before being added to the medium. These cells were confirmed to be free of mycoplasma infection, as measured by the Duke Cell Culture Facility using MycoAlert PLUS test (Lonza). S2 and KC cell lines were stably transduced with the pMKInt-Hyg vector encoding *Ras*^*V12*^*Common* and *Ras*^*V12*^*Rare* cDNAs using 1000 ng of DNA in 6 well plates per manufacturer instructions (Effectene transfection reagent, Qiagen). The following day, Schneider’s media was changed, and cells were seeded in a coated culture dish (100x20 mm). Four days later, cells were passaged with fresh Schneider’s medium and 200 μg/ ml hygromycin (Invitrogen) was added. The stably transfected cells were selected within a month growing in media containing hygromycin. Three days prior to any experiment, these cells were grown in media without hygromycin. Four million S2 cells that were stably transduced with *Ras*^*V12*^*Common* and *Ras*^*V12*^*Rare* plasmids were seeded into coated tissue culture dishes (60x15mm, VWR) with 2 ml of Schneider’s media (without FBS). Sixty micrograms of *RpS21* dsRNA were added on top of these cells. One hour later, two ml Schneider’s media containing 20% FBS were added on top of 2 ml Schneider’s media without FBS resulting in medium with 10% FBS concentration in total media of this culture. Within 16–24 hours after RNAi treatment, expression of *Ras*^*V12*^*Common* and *Ras*^*V12*^*Rare* transgenes were induced by CuSO4 for another 12 hours. Finally, these cells were collected 30–36 hours after dsRNA treatment.

#### dsRNA synthesis

S2 cell DNA was used to produce a PCR template for *RpS21* dsRNA production using the forward primer “TAATACGACTCACTATAGGGTTACTGACCAGCCGATACCC” and reverse primer “TAATACGACTCACTATAGGGCCACGCTTAGAAGTTCCTGC”. Next, 500 ng of *RpS21* PCR template was used for an *in vitro* production of dsRNA as instructed in the MEGAscrip T7 transcription kit (ThermoFisher). The dsRNA solution was cleared using MegaClear kit (ThermoFisher). Finally, the concentration of *RpS21* dsRNA was measured and stored in -80°C for future use.

#### Gene enrichment analysis and statistical analyses

To determine the Codon Adaptation Index (CAI), sequences were entered at the CAIcal web-server (http://genomes.urv.es/CAIcal [108]. For gene enrichment, deficiency sequence boundaries were defined using coordinates available through FlyBase [109] and the Bloomington *Drosophila* Stock Center website. Deficiencies were then uploaded as a custom BED track to the UCSC Genome Browser (Reference Assembly ID: dm6). Genes overlapping the deficiency coordinates were then extracted using BEDtools for additional analysis [110]. A deficiency was determined to contain known *Ras* modifiers if any of the deficiency covered genes known as *Drosophila Ras85D* genetic interactors (332 interactors, FlyBase). Enhancers and suppressor deficiencies were analyzed using the same metric against known *Ras85D* interactors of the same respective modifier type. Statistical analysis (chi-square) was performed using Graphpad Prism v8.1. FlyBase Gene Group Enrichment analysis was performed by comparing deficiency covered genes with pre-defined FlyBase Gene Groups. Analysis and statistical tests were performed in R using Gene Overlap package (https://rdrr.io/bioc/GeneOverlap/) and results are reported as adjusted p-values (False Discovery Rate [111], using Benjamini Hochberg correction). Graphs and statistical analyses were generated using GraphPad Prism 7. Statistical tests and adjusted P-values are detailed in figure legends. For all tests, adjusted P-value reporting is as follows: (P>0.05, n.s.; P<0.05,*; P<0.01,**; P<0.001,***, P<0.0001,****).

## Supporting information

S1 Fig *Drosophila Ras*^*V12*^ *Rare* more closely resembles Human KRasB than other *Drosophila Ras* transgenes. **(a)** Alignments of *Ras* transgenes. Nucleotide changes highlighted for *Ras*^*V12*^*Common* (red) and *Ras*^*V12*^*Rare* (blue). Table with overall CAI score and GC content for *Ras* transgenes. (**b)** Codon Adaptation Index (CAI) plot. Transparent circles, squares, and triangles are individual CAIs per codon. Solid lines represent a best-fit line of individual points for each transgene. **(c)** Amino acid alignment of endogenous *Drosophila Ras85D* with human KRASA and KRASB, over a region of sequence divergence between KRASA and KRASB. The percent identity is noted. **(d)** Nucleic acid alignment of the four transgenes used in this study with human KRASB. **(e)** Nucleic acid phylogenetic tree of human KRASB and the four transgenes used in this study, with the percent identity of each gene/transgene to *Drosophila Ras*^*WT*^*Native* indicated.
(TIF)

S2 Fig Codon manipulation of *Ras*^*V12*^ promotes differential MAPK signal strength levels in *Drosophila*. **(a)** Representative image of adult eye from animal expressing *sevGal4>Ras*^*WT*^ **(b)** Immunoblot detection of transgenic Ras^V12^ protein (with an anti-FLAG antibody) and αTubulin as a loading control from lysates derived from the head of flies with the indicated versions of transgenic *Ras*^*V12*^. (**c**) Quantification of protein levels at 25°C for blot in **S1B Fig**. a.u. = arbitrary units. Data represent mean ± SEM, 3 replicates, Tukey’s multiple comparisons test. (**d**) Biological replicate of serial dilution of Ras^V12^ common versus rare. 10, 20, and 30 ug of lysates derived from the heads of flies expressing the indicated versions of transgenic *Ras*^*V12*^ were immunoblotted with an anti-FLAG antibody, demonstrating differential expression of *Ras*^*V12*^ common and rare. Bottom: quantification and protein loaded. **(d)** Immunoblot detection of transgenic Ras^V12^ (with an anti-FLAG antibody), phosphorylated (p-) and total Mek and Erk, and actin as a loading control from lysates derived from **(e)** the head of flies with the indicated versions of transgenic *Ras*^*V12*^ or **(f)** S2 and KC cells stably transduced with expression vectors expressing the indicated *Ras*^*V12*^ transgenes. First lane is S2 cells without any transfection. (**g**) Levels of GTP-bound Ras^V12^ common versus rare. GTP-bound Ras from lysates derived from S2 cells stably expressing Ras^V12^ common versus rare (or no transgene as a control) were affinity captured with a Ras Binding Domain (RBD IP) and immunoblotted with an anti-FLAG antibody to detect the ectopic active portion of the expressed Ras^V12^ protein. Whole cell lysates (WCL) were immunoblotted with an anti-FLAG antibody to detect total ectopic Ras^V12^ protein and Actin as a loading control. One representative blot from multiple replicates is shown.
(TIF)

S3 Fig Known *Ras* modifiers alter phenotypes driven by codon-altered *Ras* transgenes. **(a)** Quantification of eye severity scores for *Ras* transgenes that are also heterozygous for known *Ras* modifiers. Data represent mean ± SEM, multiple replicates (using Dennett’s multiple comparison test). **(b)** The average number of flies eclosed per experiment for Rare and Common transgenes in a known *Ras* modifier background.
(TIF)

S4 Fig RpS21 negatively regulates Ras/MAPK signaling in settings of low signal output. **(a)** Genome map of *Df(2L)BSC692*. *RpS21* is highlighted in green. **(b)** Immunoblot detection of transgenic Ras^V12^ (with an anti-FLAG antibody), and actin as a loading control from lysates derived from the head of flies with the indicated versions of transgenic *Ras*^*V12*^ in either the wild-type (+/+) or mutant (*RpS21*^*03575*^/+) backgrounds, **(c)** Immunoblot detection of transgenic Ras^V12^ (with an anti-FLAG antibody),phosphorylated (p-) and total Mek and/or Erk, RpS21, and actin as a loading control from lysates derived from S2 cells stably transduced with expression vectors expressing the indicated *Ras*^*V12*^ transgenes in the absence (-) and presence (+) of *RpS21 RNAi* (Data represent two independent replicates.)
(TIF)

S5 Fig Sequence of codon-altered GFP transgenes. **(a)** Alignments of *GFP* transgenes. *GFP Common* contains all common codons. Nucleotide changes to generate rare codons in *GFP Rare* are highlighted in (blue). Table with overall CAI score and GC content for *GFP* transgenes. (**b)** Codon Adaptation Index (CAI) plot. Triangles are individual CAIs per codon. Solid lines represent a best-fit line of individual points for each transgene.
(TIF)

S1 Table Results of a genome-wide deficiency screen for modifiers of *Ras*^*V12*^ common and *Ras*^*V12*^ Rare eye phenotypes. See text for details.
(XLSX)

## References

[1] Hayashi S, Ogura Y. ERK signaling dynamics in the morphogenesis and homeostasis of Drosophila. Curr Opin Genet Dev. 2020;63:9–15. 10.1016/j.gde.2020.01.004 32145545

[2] Karim FD, Chang HC, Therrien M, Wassarman DA, Laverty T, Rubin GM. A screen for genes that function downstream of Ras1 during Drosophila eye development. Genetics. 1996;143:315–29. 8722784 10.1093/genetics/143.1.315 PMC1207264

[3] Maixner A, Hecker TP, Phan QN, Wassarman DA. A screen for mutations that prevent lethality caused by expression of activated sevenless and ras1 in the Drosophila embryo. Dev Genet. 1998;23:347–61. 10.1002/(SICI)1520-6408(1998)23:4&lt;347::AID-DVG9&gt;3.0.CO;2-C 9883586

[4] Therrien M, Morrison DK, Wong AM, Rubin GM. A genetic screen for modifiers of a kinase suppressor of Ras-dependent rough eye phenotype in Drosophila. Genetics. 2000;156:1231–42. 11063697 10.1093/genetics/156.3.1231 PMC1461306

[5] Chang HC, Rubin GM. 14-3-3ε positively regulates Ras-mediated signaling in Drosophila. Genes Dev. 1997;11:1132–9. 10.1101/gad.11.9.1132 9159394

[6] Rebay I, Chen F, Hsiao F, Kolodziej PA, Kuang BH, Laverty T, et al A genetic screen for novel components of the Ras/mitogen-activated protein kinase signaling pathway that interact with the yan gene of Drosophila identifies split ends, a new RNA recognition motif-containing protein. Genetics. 2000;154:695–712. 10655223 10.1093/genetics/154.2.695 PMC1460949

[7] Dickson BJ, van der Straten A. Dom\’\inguez M. & Hafen E. Mutations Modulating Raf signaling in Drosophila eye development. Genetics. 1996;142:163–71. 8770593 10.1093/genetics/142.1.163 PMC1206944

[8] Gaul U., Chang H., Choi T., Karim F. & Rubin G. M. Identification of ras targets using a genetic approach. *Ciba Found*. *Symp*. 176, 85–92– discussion 92–5 (1993). 10.1002/9780470514450.ch6 8299428

[9] Therrien M, Chang HC, Solomon NM, Karim FD, Wassarman DA, Rubin GM. KSR, a novel protein kinase required for RAS signal transduction. Cell. 1995;83:879–88. 10.1016/0092-8674(95)90204-x 8521512

[10] Kornfeld K. Hom D. B. & Horvitz H. R. The ksr-1 gene encodes a novel protein kinase involved in Ras-mediated signaling in C. elegans. Cell. 1995;83:903–13. 10.1016/0092-8674(95)90206-6 8521514

[11] Sundaram M, Han M, The C. elegans ksr-1 gene encodes a novel raf-related kinase involved in Ras-mediated signal transduction. Cell. 1995;83:889–901. 10.1016/0092-8674(95)90205-8 8521513

[12] Singh N. & Han M. sur-2, a novel gene, functions late in the let-60 ras-mediated signaling pathway during Caenorhabditis elegans vulval induction. *Genes Dev*. 9, 2251–2265 (1995). 10.1101/gad.9.18.2251 7557379

[13] Battu G. Hoier, E. F. & Hajnal, A. The C. elegans G-protein-coupled receptor SRA-13 inhibits RAS/MAPK signalling during olfaction and vulval development. Development. 2003;130:2567–77. 10.1242/dev.00497 12736202

[14] Berset T., Hoier E. F., Battu G., Canevascini S. & Hajnal A. Notch inhibition of RAS signaling through MAP kinase phosphatase LIP-1 during C. elegans vulval development. *Science (80-*.*)*. 291, 1055–1058 (2001). 10.1126/science.1055642 11161219

[15] Friedman A, Perrimon N. A functional RNAi screen for regulators of receptor tyrosine kinase and ERK signalling. Nature. 2006;444:230–4. 10.1038/nature05280 17086199

[16] Ashton-Beaucage D, Udell CM, Gendron P, Sahmi M, Lefrancois M, Baril C, et al A Functional Screen Reveals an Extensive Layer of Transcriptional and Splicing Control Underlying RAS/MAPK Signaling in Drosophila. PLoS Biol. 2014;12 10.1371/journal.pbio.1001809 24643257 PMC3958334

[17] Friedman A. A., Tucker G., Singh R., Yan D., Vinayagam A., Hu Y., et al. Proteomic and functional genomic landscape of receptor tyrosine kinase and ras to extracellular signal-regulated kinase signaling. *Sci*. *Signal*. 4, rs10 (2011). 10.1126/scisignal.2002029 22028469 PMC3439136

[18] Toettcher JE, Weiner OD, Lim WA. Using optogenetics to interrogate the dynamic control of signal transmission by the Ras/Erk module. Cell. 2013;155:1422–34. 10.1016/j.cell.2013.11.004 24315106 PMC3925772

[19] Wilson M. Z., Ravindran P. T., Lim W. A. & Toettcher J. E. Tracing Information Flow from Erk to Target Gene Induction Reveals Mechanisms of Dynamic and Combinatorial Control. *Mol*. *Cell* 67, 757–769.e5 (2017). 10.1016/j.molcel.2017.07.016 28826673 PMC5591080

[20] Johnson HE, Goyal Y, Pannucci NL, Shupbach T, Shvartsman SY, Toettcher JE. The Spatiotemporal Limits of Developmental Erk Signaling. Dev Cell. 2017;40:185–92. 10.1016/j.devcel.2016.12.002 28118601 PMC5289754

[21] Goyal Y, Jindal GA, Pelliccia JL, Yamaya K, Yeung E, Futran AS, et al Divergent effects of intrinsically active MEK variants on developmental Ras signaling. Nat Genet. 2017;49:465–9. 10.1038/ng.3780 28166211 PMC5621734

[22] Pershing NLK, Lampson BL, Belsky JA, Kaltenbrun E, MacAlpine DM, Counter CM. Rare codons capacitate Kras-driven de novo tumorigenesis. J Clin Invest. 2015;125:222–33. 10.1172/JCI77627 25437878 PMC4382256

[23] Ali M, Kaltenbrun E, Anderson GR, Stephens SJ, Arena S, Bardelli A, et al Codon bias imposes a targetable limitation on KRAS-driven therapeutic resistance. Nat Commun. 2017;8:15617 10.1038/ncomms15617 28593995 PMC5472712

[24] Neuman-Silberberg FS, Schejter E, Hoffmann FM, Shilo BZ. The Drosophila ras oncogenes: structure and nucleotide sequence. Cell. 1984;37:1027–33. 10.1016/0092-8674(84)90437-9 6430564

[25] Quax TEF, Claassens NJ, Söll D, van der Oost J. Codon Bias as a Means to Fine-Tune Gene Expression. Mol Cell. 2015;59:149–61. 10.1016/j.molcel.2015.05.035 26186290 PMC4794256

[26] Wang Y, Li C, Khan MRI, Wang Y, Ruan Y, Zhao B, et al An Engineered Rare Codon Device for Optimization of Metabolic Pathways. Sci Rep. 2016;6 10.1038/s41598-016-0015-2 26852704 PMC4745014

[27] Lampson BL, Pershing NLK, Prinz JA, Lacsina JR, Marzluff WF, Nicchitta CV, et al Rare Codons Regulate KRas Oncogenesis. Curr Biol. 2013;23:70–5. 10.1016/j.cub.2012.11.031 23246410 PMC3567844

[28] Li S, Balmain A, Counter CM. A model for RAS mutation patterns in cancers: finding the sweet spot. Nat Rev Cancer. 2018;18:767–77. 10.1038/s41568-018-0076-6 30420765

[29] Ikemura T. Codon usage and tRNA content in unicellular and multicellular organisms. Mol Biol Evol. 1985;2:13–34. 10.1093/oxfordjournals.molbev.a040335 3916708

[30] Sablok G, Nayak KC, Vazquez F, Tatarinova TV. Synonymous codon usage, GC 3, and evolutionary patterns across plastomes of three pooid model species: Emerging grass genome models for monocots. Mol Biotechnol. 2011;49:116–28. 10.1007/s12033-011-9383-9 21308422

[31] Moriyama EN, Powell JR. Codon usage bias and tRNA abundance in Drosophila. J Mol Evol. 1997;45:514–23. 10.1007/pl00006256 9342399

[32] Urrutia AO, Hurst LD. Codon usage bias covaries with expression breadth and the rate of synonymous evolution in humans, but this is not evidence for selection. Genetics. 2001. 10.1093/genetics/159.3.1191 PMC1461876 11729162

[33] Yang Z, Nielsen R. Mutation-selection models of codon substitution and their use to estimate selective strengths on codon usage. Mol Biol Evol. 2008;25:568–79. 10.1093/molbev/msm284 18178545

[34] Hense W, Anderson N, Hutter S. Stephan., W., Parsch, J., Carlini, D.B. Experimentally increased codon bias in the Drosophila Adh gene leads to an increase in larval, but not adult, alcohol dehydrogenase activity. Genetics. 2010;184:547–55. 10.1534/genetics.109.111294 19966063 PMC2828731

[35] Burow DA, Martin S, Quail JF, Alhusaini N, Coller J, Cleary MD. Attenuated Codon Optimality Contributes to Neural-Specific mRNA Decay in Drosophila. Cell Rep. 2018;24:1704–12. 10.1016/j.celrep.2018.07.039 30110627 PMC6169788

[36] Plotkin JB, Kudla G. Synonymous but not the same: the causes and consequences of codon bias. Nat Rev Genet. 2011;12:32–42. 10.1038/nrg2899 21102527 PMC3074964

[37] Hanson G, Coller J. Translation and Protein Quality Control: Codon optimality, bias and usage in translation and mRNA decay. Nat Rev Mol Cell Biol. 2018;19:20–30. 10.1038/nrm.2017.91 29018283 PMC6594389

[38] Sharp PM, Li WH. The codon adaptation index-a measure of directional synonymous codon usage bias, and its potential applications. Nucleic Acids Res. 1987;15:1281–95. 10.1093/nar/15.3.1281 3547335 PMC340524

[39] Fortini ME, Simon MA, Rubin GM. Signalling by the sevenless protein tyrosine kinase is mimicked by Ras1 activation. Nature. 1992;355:559–61. 10.1038/355559a0 1311054

[40] Gaul U, Mardon G, Rubin GM. A putative Ras GTPase activating protein acts as a negative regulator of signaling by the Sevenless receptor tyrosine kinase. Cell. 1992;68:1007–19. 10.1016/0092-8674(92)90073-l 1547500

[41] Biggs WH, Zavitz KH, Dickson B, van der Straten A, Brunner D, Hafen E, et al The Drosophila rolled locus encodes a MAP kinase required in the sevenless signal transduction pathway. EMBO J. 1994;13:1628–35. 8157002 10.1002/j.1460-2075.1994.tb06426.x PMC394993

[42] De Rooij J, Bos JL. Minimal Ras-binding domain of Raf1 can be used as an activation-specific probe for Ras. Oncogene. 1997;14:623–5. 10.1038/sj.onc.1201005 9053862

[43] Ikemura T. Correlation between the abundance of Escherichia coli transfer RNAs and the occurrence of the respective codons in its protein genes: A proposal for a synonymous codon choice that is optimal for the E coli translational system *J Mol Biol*. 1981;151:389–409. 10.1016/0022-2836(81)90003-6 6175758

[44] Spanjaard RA, Van Duin J. Translation of the sequence AGG-AGG yields 50% ribosomal frameshift. Proc Natl Acad Sci U S A. 1988;85:7967–71. 10.1073/pnas.85.21.7967 3186700 PMC282334

[45] Kramer E. B. & Farabaugh, P. J. The frequency of translational misreading errors in E. coli is largely determined by tRNA competition. RNA. 2007;13:87–96. 10.1261/rna.294907 17095544 PMC1705757

[46] Rosenberg AH, Goldman E, Dunn JJ, Studier FW, Zubay G. Effects of consecutive AGG codons on translation in Escherichia coli, demonstrated with a versatile codon test system. J Bacteriol. 1993;175:716–22. 10.1128/jb.175.3.716-722.1993 7678594 PMC196210

[47] Chu D, Kazana E, Bellanger N, Singh T, Tuite MF, von der Haar T. Translation elongation can control translation initiation on eukaryotic mRNAs. EMBO J. 2014;33:21–34. 10.1002/embj.201385651 24357599 PMC3990680

[48] Fu J, Dang Y, Counter C, Liu Y. Codon usage regulates human KRAS expression at both transcriptional and translational levels. J Biol Chem. 2018;293:17929–40. 10.1074/jbc.RA118.004908 30275015 PMC6240855

[49] Zhoua Z, Dang Y, Zhou M, Li L, Yu CH, Fu J, et al Codon usage is an important determinant of gene expression levels largely through its effects on transcription. Proc Natl Acad Sci U S A. 2016;113:E6117–25. 10.1073/pnas.1606724113 27671647 PMC5068308

[50] Harigaya Y, Parker R. Analysis of the association between codon optimality and mRNA stability in Schizosaccharomyces pombe. BMC Genomics. 2016;17 10.1186/s12864-015-2333-3 27825301 PMC5101800

[51] Bazzini AA, Del Viso F, Moreno-Mateos MA, Johnstone TG, Vejnar CE, Qin Y, et al Codon identity regulates mRNA stability and translation efficiency during the maternal-to-zygotic transition. EMBO J. 2016;35:2087–103. 10.15252/embj.201694699 27436874 PMC5048347

[52] Mishima Y, Tomari Y. Codon Usage and 3’ UTR Length Determine Maternal mRNA Stability in Zebrafish. Mol Cell. 2016;61:874–85. 10.1016/j.molcel.2016.02.027 26990990

[53] Radhakrishnan A., Chen Y.H., Martin S., Alhusaini N., Green R., Coller J. The DEAD-Box Protein Dhh1p Couples mRNA Decay and Translation by Monitoring Codon Optimality. *Cell* 167, 122–132.e9 (2016). 10.1016/j.cell.2016.08.053 27641505 PMC5635654

[54] Struhl G, Basler K. Organizing activity of wingless protein in Drosophila. Cell. 1993;72:527–40. 10.1016/0092-8674(93)90072-x 8440019

[55] Karim FD, Rubin GM. Ectopic expression of activated Ras1 induces hyperplastic growth and increased cell death in Drosophila imaginal tissues. Development. 1998;125:1–9. 9389658 10.1242/dev.125.1.1

[56] Jiang Y, Scott KL, Kwak SJ, Chen R, Mardon G. Sds22/PP1 links epithelial integrity and tumor suppression via regulation of myosin II and JNK signaling. Oncogene. 2011;30:3248–60. 10.1038/onc.2011.46 21399659 PMC3141090

[57] Shen J, Curtis C, Tavaré S, Tower J. A screen of apoptosis and senescence regulatory genes for life span effects when over-expressed in Drosophila. Aging (Albany NY). 2009;1 (191–211). 10.18632/aging.100018 20157509 PMC2806004

[58] Cox AD, Der CJ. The dark side of Ras: Regulation of apoptosis. Oncogene. 2003;22:8999–9006. 10.1038/sj.onc.1207111 14663478

[59] Karnoub AE, Weinberg RA. Ras oncogenes: Split personalities. Nat Rev Mol Cell Biol. 2008;9:517–31. 10.1038/nrm2438 18568040 PMC3915522

[60] Rebay I, Rubin GM. Yan functions as a general inhibitor of differentiation and is negatively regulated by activation of the Ras1/MAPK pathway. Cell. 1995;81:857–66. 10.1016/0092-8674(95)90006-3 7781063

[61] Lai ZC, Rubin GM. Negative control of photoreceptor development in Drosophila by the product of the yan gene, an ETS domain protein. Cell. 1992;70:609–20. 10.1016/0092-8674(92)90430-k 1505027

[62] Ray M, Lakhotia SC. The commonly used eye-specific sev-GAL4 and GMR-GAL4 drivers in Drosophila melanogaster are expressed in tissues other than eyes also. J Genet. 2015;94:407–16. 10.1007/s12041-015-0535-8 26440079

[63] Rørth P. A modular misexpression screen in Drosophila detecting tissue-specific phenotypes. Proc Natl Acad Sci U S A. 1996;93:12418–22. 10.1073/pnas.93.22.12418 8901596 PMC38006

[64] Cook RK, Christensen SJ, Deal JA, Coburn RA, Deal ME, Gresens JM, et al The generation of chromosomal deletions to provide extensive coverage and subdivision of the Drosophila melanogaster genome. Genome Biol. 2012;13:R21 10.1186/gb-2012-13-3-r21 22445104 PMC3439972

[65] Török I, Hermann-Horle D, Kiss I, Tick G, Speer G, Schmitt R, et al Down-regulation of RpS21, a putative translation initiation factor interacting with P40, produces viable minute imagos and larval lethality with overgrown hematopoietic organs and imaginal discs. Mol Cell Biol. 1999;19:2308–21. 10.1128/mcb.19.3.2308 10022917 PMC84023

[66] Nilson LA, Schüpbach T, Schüpbach T. 7 EGF Receptor Signaling in Drosophila Oogenesis. Curr Top Dev Biol. 1998;44:203–43. 10.1016/s0070-2153(08)60471-8 9891881

[67] Cheung LS, Schüpbach T, Shvartsman SY. Pattern formation by receptor tyrosine kinases: Analysis of the Gurken gradient in Drosophila oogenesis. Curr Opin Genet Dev. 2011;21:719–25. 10.1016/j.gde.2011.07.009 21862318 PMC6178945

[68] Halfar K, Rommel C, Stocker H, Hafen E. Ras controls growth, survival and differentiation in the Drosophila eye by different thresholds of MAP kinase activity. Development. 2001;128:1687–96. 11290305 10.1242/dev.128.9.1687

[69] Sears R, Nuckolis F, Haura E, Taya Y, Tamai K, Nevins JR. Multiple Ras-dependent phosphorylation pathways regulate Myc protein stability. Genes Dev. 2000;14:2501–14. 10.1101/gad.836800 11018017 PMC316970

[70] Magudia K, Lahoz A, Hall A. K-Ras and B-Raf oncogenes inhibit colon epithelial polarity establishment through up-regulation of c-myc. J Cell Biol. 2012;198:185–94. 10.1083/jcb.201202108 22826122 PMC3410422

[71] Tsai W. B., Aiba I., Long Y., Lin H.K., Feun L., Savaraj, et al. Activation of Ras/PI3K/ERK pathway induces c-Myc stabilization to upregulate argininosuccinate synthetase, leading to arginine deiminase resistance in melanoma cells. *Cancer Res*. 72, 2622–2633 (2012). 10.1158/0008-5472.CAN-11-3605 22461507 PMC3433038

[72] Prober DA, Edgar BA. Interactions between Ras1, dMyc, and dPI3K signaling in the developing Drosophila wing. Genes Dev. 2002;16:2286–99. 10.1101/gad.991102 12208851 PMC186666

[73] Rauen K, The A. RASopathies. Annu Rev Genomics Hum Genet. 2013;14:355–69. 10.1146/annurev-genom-091212-153523 23875798 PMC4115674

[74] Prior IA, Lewis PD, Mattos C. A comprehensive survey of Ras mutations in cancer. Cancer Res. 2012;72:2457–67. 10.1158/0008-5472.CAN-11-2612 22589270 PMC3354961

[75] Bridges C. B. & Morgan T. H. *The third-chromosome group of mutant characters of Drosophila melanogaster*,. *The third-chromosome group of mutant characters of Drosophila melanogaster*, (Carnegie Institution of Washington, 2011). 10.5962/bhl.title.24013

[76] Schultz J. The Minute Reaction in the Development of DROSOPHILA MELANOGASTER. Genetics. 1929;14:366–419. 17246581 10.1093/genetics/14.4.366 PMC1201041

[77] Marygold SJ, Roote J, Reuter G, Lambertsson A, Ashburner M, Millburn GH, et al The ribosomal protein genes and Minute loci of Drosophila melanogaster. Genome Biol. 2007;8 10.1186/gb-2007-8-10-r216 17927810 PMC2246290

[78] Duttagupta A. K. & Shellenbarger D. L. Genetics of Minute Locus in Drosophila Melanogaster in *Development and Neurobiology of Drosophila* 25–33 (Springer US, 1980). 10.1007/978-1-4684-7968-3_3 6779793

[79] Watson KL, Johnson TK, Denell RE. Lethal(1)aberrant immune response mutations leading to melanotic tumor formation in Drosophila melanogaster. Dev Genet. 1991;12:173–87. 10.1002/dvg.1020120302 1907895

[80] Watson KL, Konrad KD, Woods DF, Bryant PJ. Drosophila homolog of the human S6 ribosomal protein is required for tumor suppression in the hematopoietic system. Proc Natl Acad Sci U S A. 1992;89:11302–6. 10.1073/pnas.89.23.11302 1454811 PMC50538

[81] Stewart MJ, Denell R. Mutations in the Drosophila gene encoding ribosomal protein S6 cause tissue overgrowth. Mol Cell Biol. 1993;13:2524–35. 10.1128/mcb.13.4.2524 8384310 PMC359579

[82] Marygold SJ, Coelho CMA, Leevers SJ. Genetic analysis of RpL38 and RpL5, two minute genes located in the centric heterochromatin of chromosome 2 of Drosophila melanogaster. Genetics. 2005;169:683–95. 10.1534/genetics.104.034124 15520262 PMC1449105

[83] Lin JI, Mitchell NC, Kalcina M, Tchobubrieva E, Stewart MJ, Marygold SJ, et al Drosophila ribosomal protein mutants control tissue growth non-autonomously via effects on the prothoracic gland and ecdysone. PLoS Genet. 2011;7 10.1371/journal.pgen.1002408 22194697 PMC3240600

[84] Amsterdam A, Sadler KC, Lai K, Farrington S, Bronson RT, Lees JA, et al Many ribosomal protein genes are cancer genes in zebrafish. PLoS Biol. 2004;2 10.1371/journal.pbio.0020139 15138505 PMC406397

[85] Ajore R, Raiser D, McConkey M, Joud M, Boidol B, Mar B, et al Deletion of ribosomal protein genes is a common vulnerability in human cancer, especially in concert with TP 53 mutations. EMBO Mol Med. 2017;9:498–507. 10.15252/emmm.201606660 28264936 PMC5376749

[86] Russo A. & Russo G. Ribosomal proteins control or bypass p53 during nucleolar stress. *International Journal of Molecular Sciences* vol. 18 (2017). 10.3390/ijms18010140 28085118 PMC5297773

[87] Chen J, Guo K, Kastan MB. Interactions of nucleolin and ribosomal protein L26 (RPL26) in translational control of human p53 mRNA. J Biol Chem. 2012;287:16467–76. 10.1074/jbc.M112.349274 22433872 PMC3351294

[88] Sloan KE, Bohnsack MT, Watkins NJ. The 5S RNP Couples p53 Homeostasis to Ribosome Biogenesis and Nucleolar Stress. Cell Rep. 2013;5:237–47. 10.1016/j.celrep.2013.08.049 24120868 PMC3808153

[89] Donati G, Peddigari S, Mercer CA, Thomas G. 5S Ribosomal RNA Is an Essential Component of a Nascent Ribosomal Precursor Complex that Regulates the Hdm2-p53 Checkpoint. Cell Rep. 2013;4:87–98. 10.1016/j.celrep.2013.05.045 23831031 PMC3928573

[90] Wan F, Anderson DE, Barnitz RA, Snow A, Bidere N, Zheng L, et al Ribosomal Protein S3: A KH Domain Subunit in NF-κB Complexes that Mediates Selective Gene Regulation. Cell. 2007;131:927–39. 10.1016/j.cell.2007.10.009 18045535

[91] Donati G, Brighenti E, Vici M, Mazzini G, Trere D, Montanaro L, et al Selective inhibition of rrna transcription downregulates E2F-1: A new p53-independent mechanism linking cell growth to cell proliferation. J Cell Sci. 2011;124:3017–28. 10.1242/jcs.086074 21878508

[92] Barna M, Pusic A, Zollo O, Costa M, Kondrashov N, Rego E, et al Suppression of Myc oncogenic activity by ribosomal protein haploinsufficiency. Nature. 2008;456:971–5. 10.1038/nature07449 19011615 PMC2880952

[93] Lessard F, Igelmann S, Trahan C, Huot G, Saint-Germain E, Mignacca L, et al Senescence-associated ribosome biogenesis defects contributes to cell cycle arrest through the Rb pathway. Nat Cell Biol. 2018;20:789–99. 10.1038/s41556-018-0127-y 29941930

[94] Wan Y, Zhang Q, Zhang Z, Song B, Wang X, Zhang Y, et al Transcriptome analysis reveals a ribosome constituents disorder involved in the RPL5 downregulated zebrafish model of Diamond-Blackfan anemia. BMC Med Genet. 2016;9 10.1186/s12881-016-0273-7 26961822 PMC4785739

[95] Mirabello L, Khincha PP, Ellis SR, Giri N, Brodie S, Chanrasekharappa SC, et al Novel and known ribosomal causes of Diamond-Blackfan anaemia identified through comprehensive genomic characterisation. J Med Genet. 2017;54:417–25. 10.1136/jmedgenet-2016-104346 28280134 PMC12265046

[96] Horos R, Ijspeert H, Popisilova D, Sendtner R, Andrieu-Soler C, Taskesen E, et al Ribosomal deficiencies in Diamond-Blackfan anemia impair translation of transcripts essential for differentiation of murine and human erythroblasts. Blood. 2012;119:262–72. 10.1182/blood-2011-06-358200 22058113

[97] Farrar JE, Vlachos A, Atsidaftos E, Carlson-Donohoe H, Markello TC, Arceci RJ, et al Ribosomal protein gene deletions in Diamond-Blackfan anemia. Blood. 2011;118:6943–51. 10.1182/blood-2011-08-375170 22045982 PMC3245214

[98] Shi Z., Fujii K., Kovary K.M., Genuth N.R., Rost H.L., Teruel M.N., et al. Heterogeneous Ribosomes Preferentially Translate Distinct Subpools of mRNAs Genome-wide. *Mol*. *Cell* 67, 71–83.e7 (2017). 10.1016/j.molcel.2017.05.021 28625553 PMC5548184

[99] Lessard F., Brakier-Gingras L. & Ferbeyre G. Ribosomal Proteins Control Tumor Suppressor Pathways in Response to Nucleolar Stress. *BioEssays* vol. 41 (2019). 10.1002/bies.201800183 30706966

[100] Warner JR, McIntosh KB. How Common Are Extraribosomal Functions of Ribosomal Proteins? Mol Cell. 2009;34:3–11. 10.1016/j.molcel.2009.03.006 19362532 PMC2679180

[101] Dionne KL, Bergeron D, Landry-Voyer AM, Bachand F. The 40S ribosomal protein uS5 (RPS2) assembles into an extraribosomal complex with human ZNF277 that competes with the PRMT3– uS5 interaction. J Biol Chem. 2019;294:1944–55. 10.1074/jbc.RA118.004928 30530495 PMC6369276

[102] Simsek D., Tiu G.C., Flynn R.A., Byeon G.W., Leppek K., Xu A.F., et al. The Mammalian Ribo-interactome Reveals Ribosome Functional Diversity and Heterogeneity. *Cell* 169, 1051–1065.e18 (2017). 10.1016/j.cell.2017.05.022 28575669 PMC5548193

[103] Xue S, Tian S, Fujii K, Kladwang W, Das R, Barna M. RNA regulons in Hox 5[prime] UTRs confer ribosome specificity to gene regulation. Nature. 2015;517:33–8. 10.1038/nature14010 25409156 PMC4353651

[104] Wang T, Wang ZY, Zeng LY, Gao YZ, Yan YX, Zhang Q. Down-regulation of ribosomal protein RPS21 inhibits invasive behavior of osteosarcoma cells through the inactivation of MAPK pathway. Cancer Manag Res. 2020;12:4949–55. 10.2147/CMAR.S246928 32612383 PMC7323807

[105] Yanagawa SI, Lee JS, Ishimoto A. Identification and characterization of a novel line of Drosophila Schneider s2 cells that respond to wingless signaling. J Biol Chem. 1999;273:32353–9. 10.1074/jbc.273.48.32353 9822716

[106] Wang J-W, Beck ES, McCabe BD. A modular toolset for recombination transgenesis and neurogenetic analysis of Drosophila. PLoS One. 2012;7:e42102 10.1371/journal.pone.0042102 22848718 PMC3405054

[107] Stormo BM, Fox DT. Distinct responses to reduplicated chromosomes require distinct Mad2 responses. elife. 2016;5 10.7554/eLife.15204 27159240 PMC4898934

[108] Puigbò P, Bravo IG, Garcia-Vallve S. CAIcal: A combined set of tools to assess codon usage adaptation. Biol Direct. 2008;3 10.1186/1745-6150-3-3 18796141 PMC2553769

[109] Thurmond J., Goodman J.L., Strelets V.B., Attrill H., Gramates L.S., Marygold S.J., et al. FlyBase 2.0: The next generation. *Nucleic Acids Res*. 47, D759–D765 (2019). 10.1093/nar/gky1003 30364959 PMC6323960

[110] Quinlan AR, Hall IM. BEDTools: A flexible suite of utilities for comparing genomic features. Bioinformatics. 2010;26:841–2. 10.1093/bioinformatics/btq033 20110278 PMC2832824

[111] Benjamini Y, Hochberg Y. Controlling the False Discovery Rate: A Practical and Powerful Approach to Multiple Testing. J R Stat Soc Ser B. 1995;57:289–300.

