# Supplementary figures and images for: Exploiting codon usage identifies intensity-specific modifiers of Ras/MAPK signaling in vivo
Source: PLoS Genet. 2020 Dec 9;16(12):e1009228. doi: 10.1371/journal.pgen.1009228 (PMC7752094; doi:10.1371/journal.pgen.1009228)

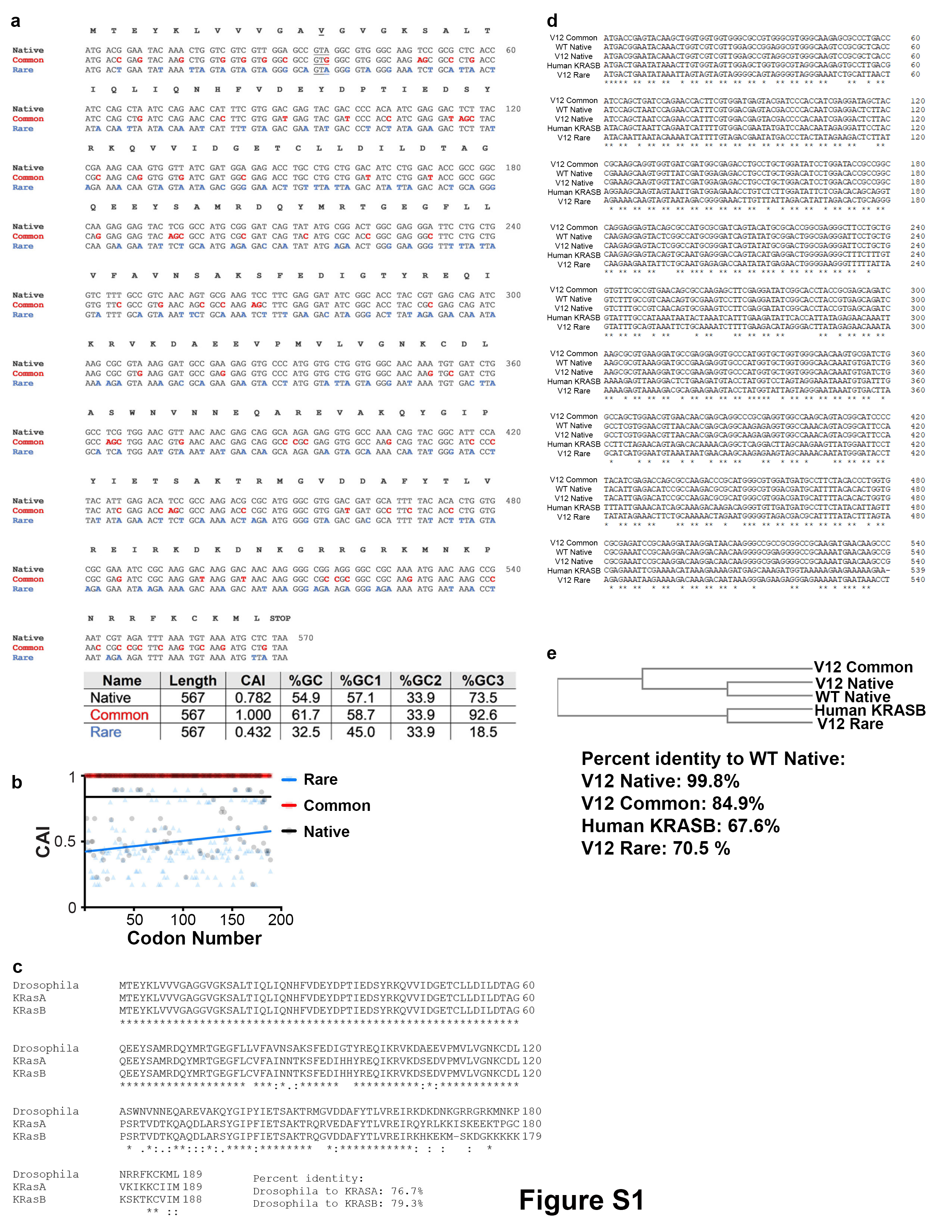

Supplement: S1 Fig — (a) Alignments of Ras transgenes. Nucleotide changes highlighted for RasV12Common (red) and RasV12Rare (blue). Table with overall CAI score and GC content for Ras transgenes. (b) Codon Adaptation Index (CAI) plot. Transparent circles, squares, and triangles are individual CAIs per codon. Solid lines represent a best-fit line of individual points for each transgene. (c) Amino acid alignment of endogenous Drosophila Ras85D with human KRASA and KRASB, over a region of sequence divergence between KRASA and KRASB. The percent identity is noted. (d) Nucleic acid alignment of the four transgenes used in this study with human KRASB. (e) Nucleic acid phylogenetic tree of human KRASB and the four transgenes used in this study, with the percent identity of each gene/transgene to Drosophila RasWTNative indicated. (TIF) [file pgen.1009228.s001.tif]

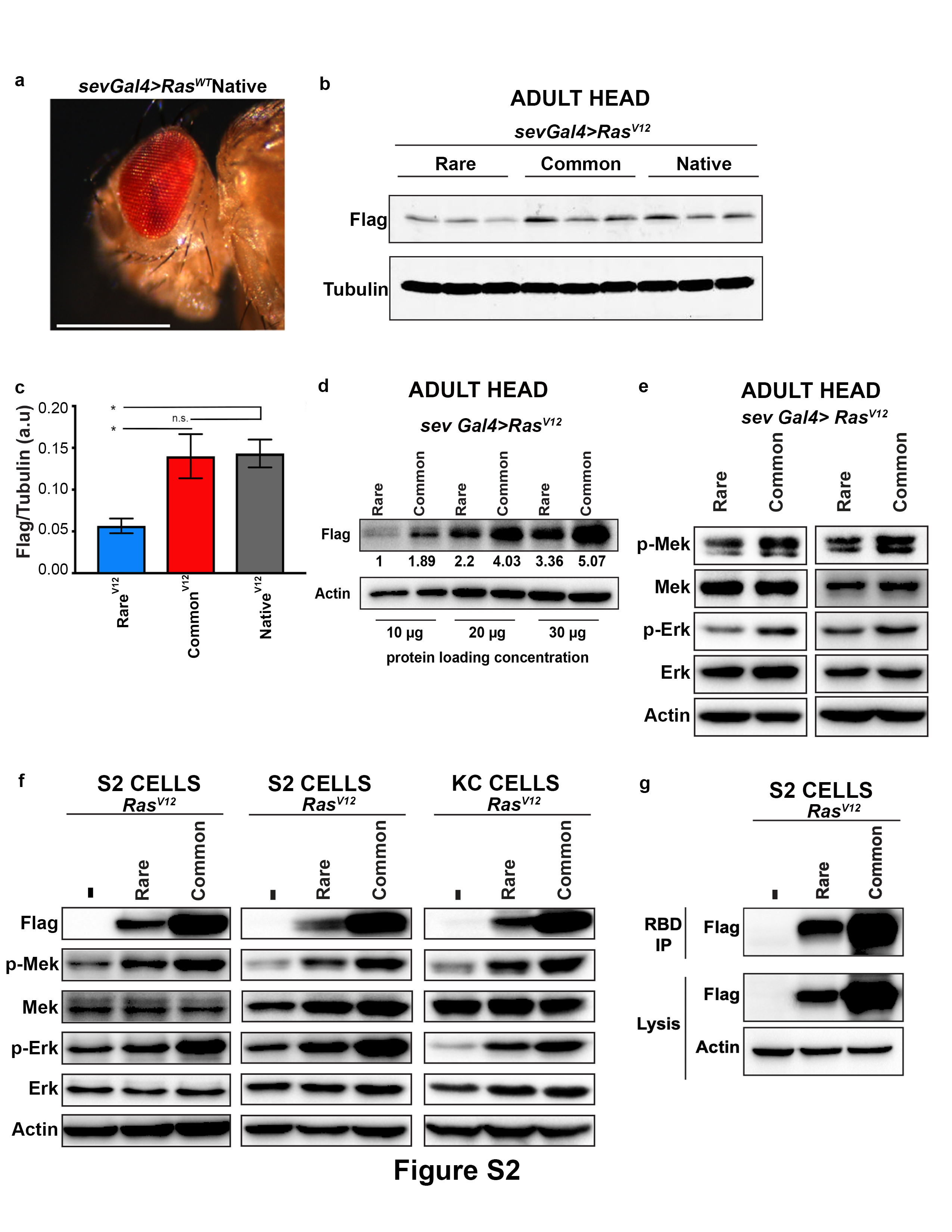

Supplement: S2 Fig — (a) Representative image of adult eye from animal expressing sevGal4>RasWT (b) Immunoblot detection of transgenic RasV12 protein (with an anti-FLAG antibody) and αTubulin as a loading control from lysates derived from the head of flies with the indicated versions of transgenic RasV12. (c) Quantification of protein levels at 25°C for blot in S1B Fig. a.u. = arbitrary units. Data represent mean ± SEM, 3 replicates, Tukey’s multiple comparisons test. (d) Biological replicate of serial dilution of RasV12 common versus rare. 10, 20, and 30 ug of lysates derived from the heads of flies expressing the indicated versions of transgenic RasV12 were immunoblotted with an anti-FLAG antibody, demonstrating differential expression of RasV12 common and rare. Bottom: quantification and protein loaded. (d) Immunoblot detection of transgenic RasV12 (with an anti-FLAG antibody), phosphorylated (p-) and total Mek and Erk, and actin as a loading control from lysates derived from (e) the head of flies with the indicated versions of transgenic RasV12 or (f) S2 and KC cells stably transduced with expression vectors expressing the indicated RasV12 transgenes. First lane is S2 cells without any transfection. (g) Levels of GTP-bound RasV12 common versus rare. GTP-bound Ras from lysates derived from S2 cells stably expressing RasV12 common versus rare (or no transgene as a control) were affinity captured with a Ras Binding Domain (RBD IP) and immunoblotted with an anti-FLAG antibody to detect the ectopic active portion of the expressed RasV12 protein. Whole cell lysates (WCL) were immunoblotted with an anti-FLAG antibody to detect total ectopic RasV12 protein and Actin as a loading control. One representative blot from multiple replicates is shown. (TIF) [file pgen.1009228.s002.tif]

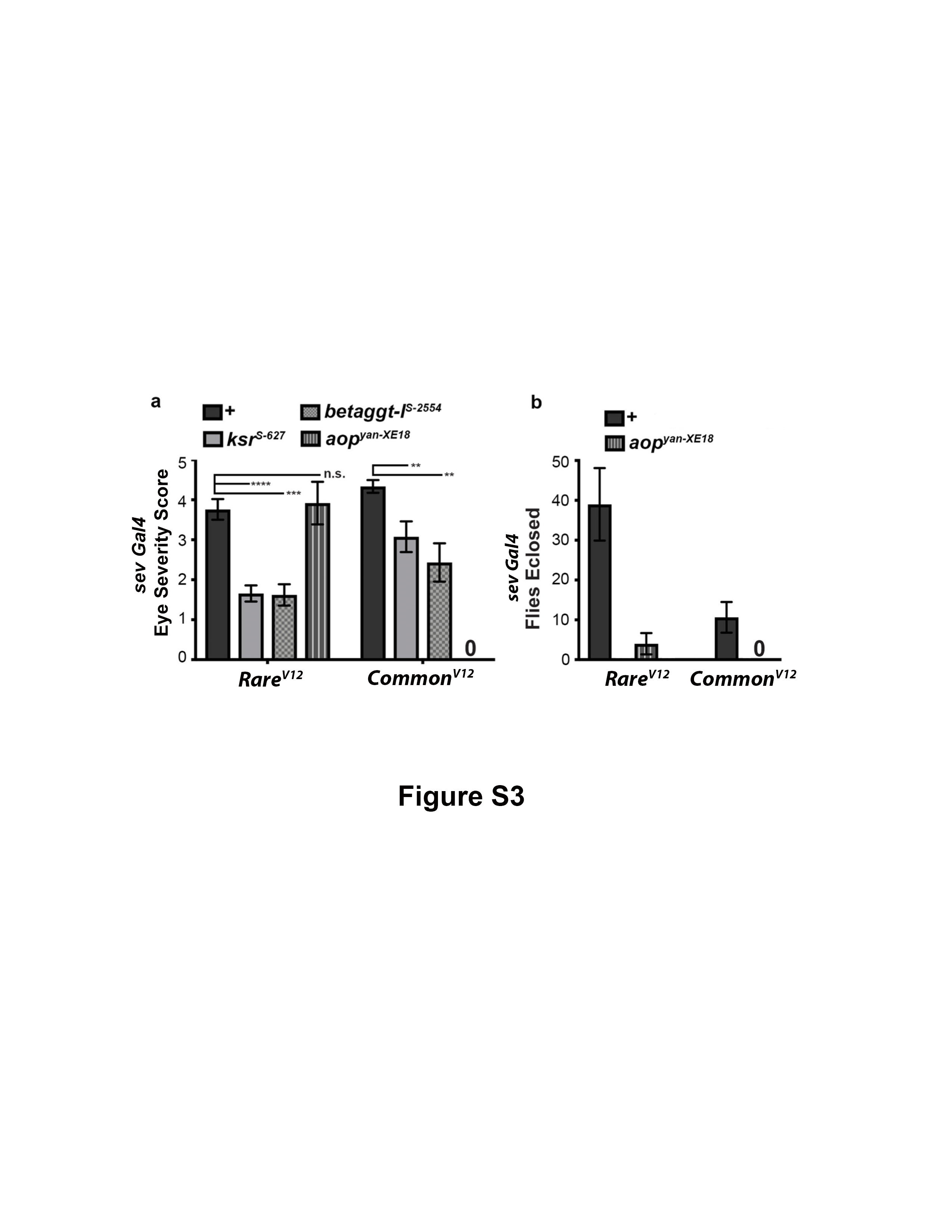

Supplement: S3 Fig — (a) Quantification of eye severity scores for Ras transgenes that are also heterozygous for known Ras modifiers. Data represent mean ± SEM, multiple replicates (using Dennett’s multiple comparison test). (b) The average number of flies eclosed per experiment for Rare and Common transgenes in a known Ras modifier background. (TIF) [file pgen.1009228.s003.tif]

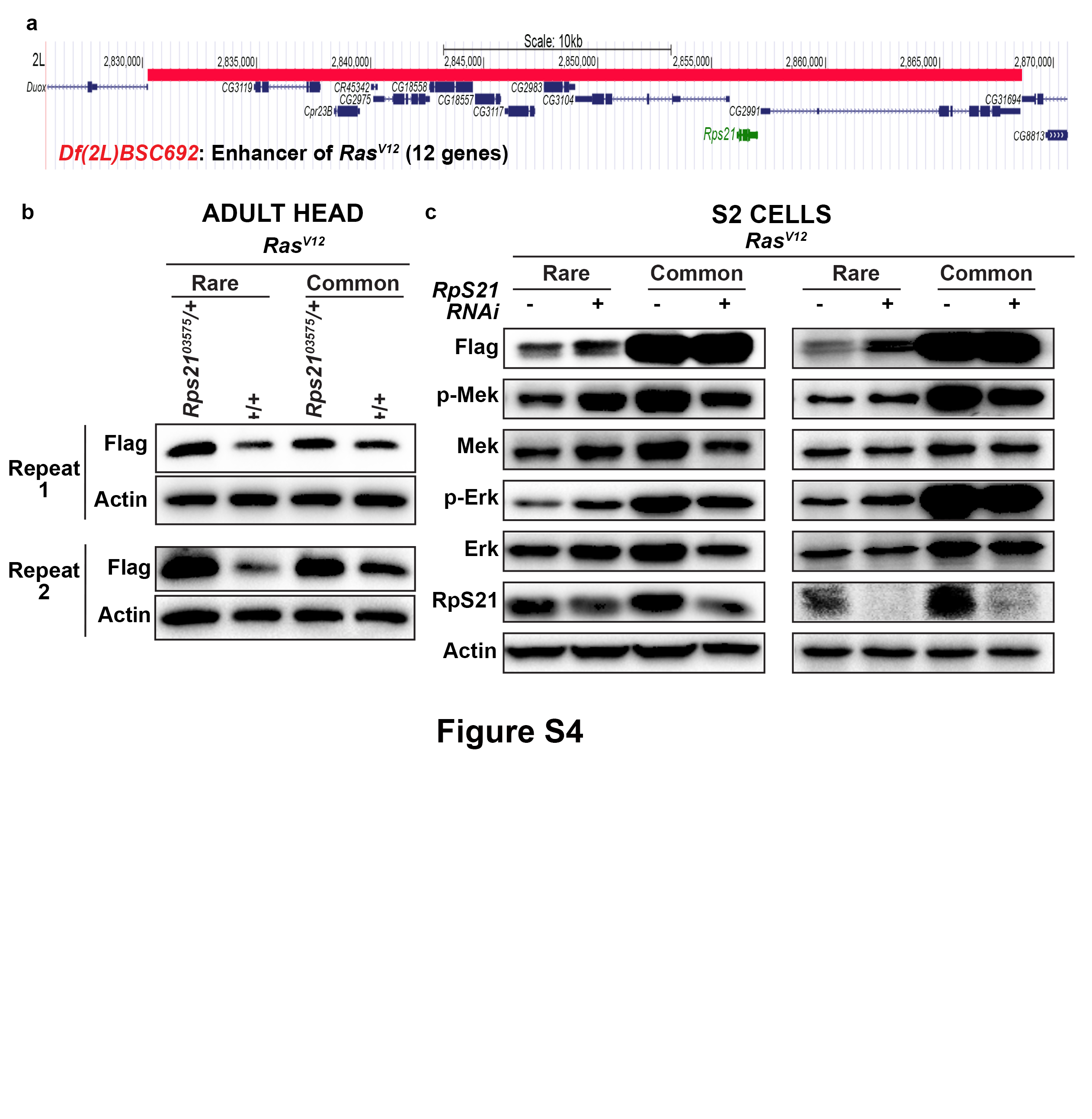

Supplement: S4 Fig — (a) Genome map of Df(2L)BSC692. RpS21 is highlighted in green. (b) Immunoblot detection of transgenic RasV12 (with an anti-FLAG antibody), and actin as a loading control from lysates derived from the head of flies with the indicated versions of transgenic RasV12 in either the wild-type (+/+) or mutant (RpS2103575/+) backgrounds, (c) Immunoblot detection of transgenic RasV12 (with an anti-FLAG antibody),phosphorylated (p-) and total Mek and/or Erk, RpS21, and actin as a loading control from lysates derived from S2 cells stably transduced with expression vectors expressing the indicated RasV12 transgenes in the absence (-) and presence (+) of RpS21 RNAi (Data represent two independent replicates.) (TIF) [file pgen.1009228.s004.tif]

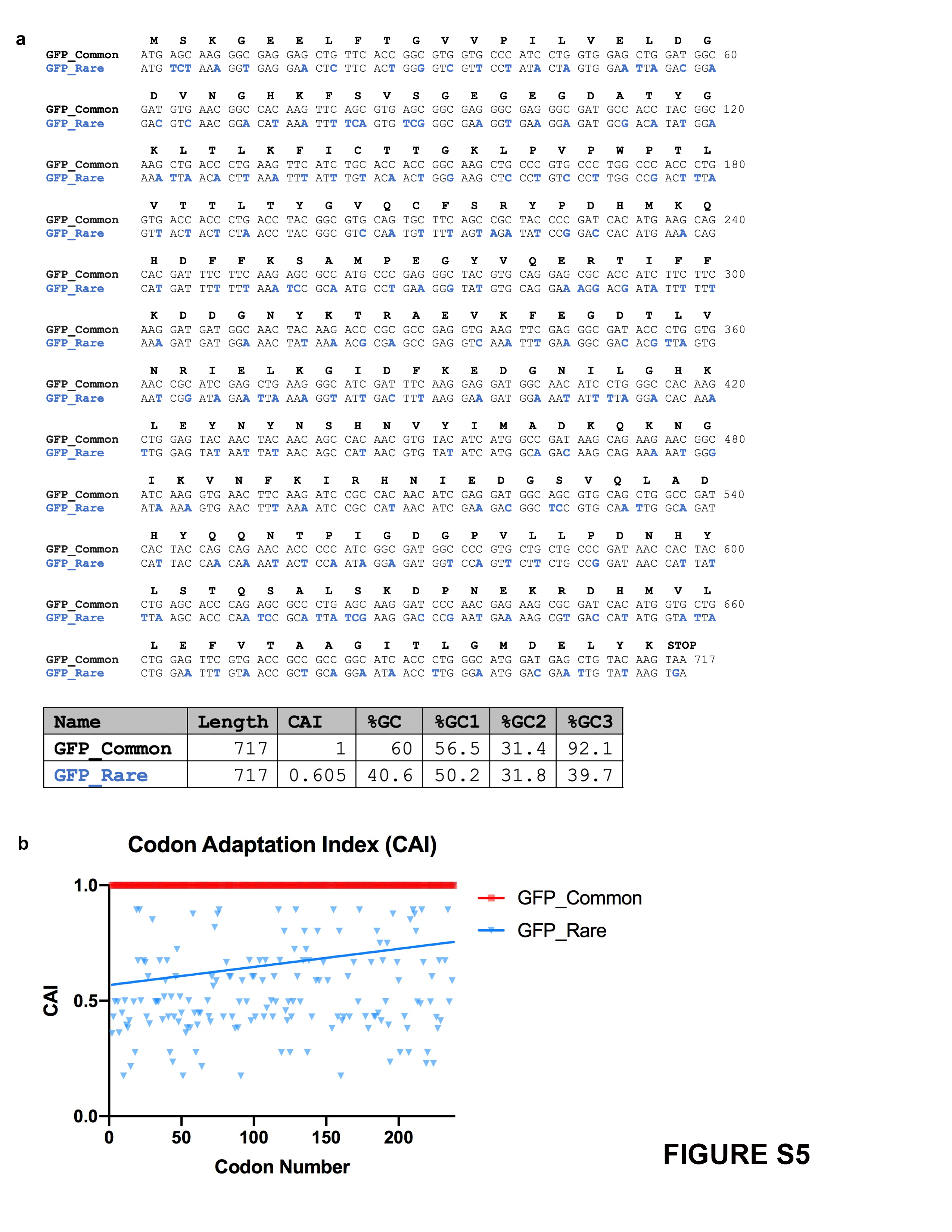

Supplement: S5 Fig — (a) Alignments of GFP transgenes. GFP Common contains all common codons. Nucleotide changes to generate rare codons in GFP Rare are highlighted in (blue). Table with overall CAI score and GC content for GFP transgenes. (b) Codon Adaptation Index (CAI) plot. Triangles are individual CAIs per codon. Solid lines represent a best-fit line of individual points for each transgene. (TIF) [file pgen.1009228.s005.tif]
